# Near-infrared photodynamic and photothermal co-therapy based on organic small molecular dyes

**DOI:** 10.1186/s12951-023-02111-x

**Published:** 2023-09-27

**Authors:** Shuang Guo, Dongyu Gu, Yi Yang, Jing Tian, Xiaoyuan Chen

**Affiliations:** 1 School of Light Industry and Chemical Engineering, Dalian Polytechnic University, Dalian, 116034 China; 2 College of Marine Science and Environment, Dalian Ocean University, Dalian, 116023 China; 3 School of Biological Engineering, Dalian Polytechnic University, Dalian, 116034 China; 4 Yong Loo Lin School of Medicine, Faculty of Engineering, National University of Singapore, Singapore, 117597 Singapore

**Keywords:** Organic small molecule dyes, Nanoplatforms, Photodynamic and photothermal co-therapy, Image-guided therapy, Near-infrared irradiation

## Abstract

Near-infrared (NIR) organic small molecule dyes (OSMDs) are effective photothermal agents for photothermal therapy (PTT) due to their advantages of low cost and toxicity, good biodegradation, and strong NIR absorption over a wide wavelength range. Nevertheless, OSMDs have limited applicability in PTT due to their low photothermal conversion efficiency and inadequate destruction of tumor regions that are nonirradiated by NIR light. However, they can also act as photosensitizers (PSs) to produce reactive oxygen species (ROS), which can be further eradicated by using ROS-related therapies to address the above limitations of PTT. In this review, the synergistic mechanism, composition, and properties of photodynamic therapy (PDT)–PTT nanoplatforms were comprehensively discussed. In addition, some specific strategies for further improving the combined PTT and PDT based on OSMDs for cancer to completely eradicate cancer cells were outlined. These strategies include performing image-guided co-therapy, enhancing tumor infiltration, increasing H_2_O_2_ or O_2_ in the tumor microenvironment, and loading anticancer drugs onto nanoplatforms to enable combined therapy with phototherapy and chemotherapy. Meanwhile, the intriguing prospects and challenges of this treatment modality were also summarized with a focus on the future trends of its clinical application.

**Graphical Abstract:**

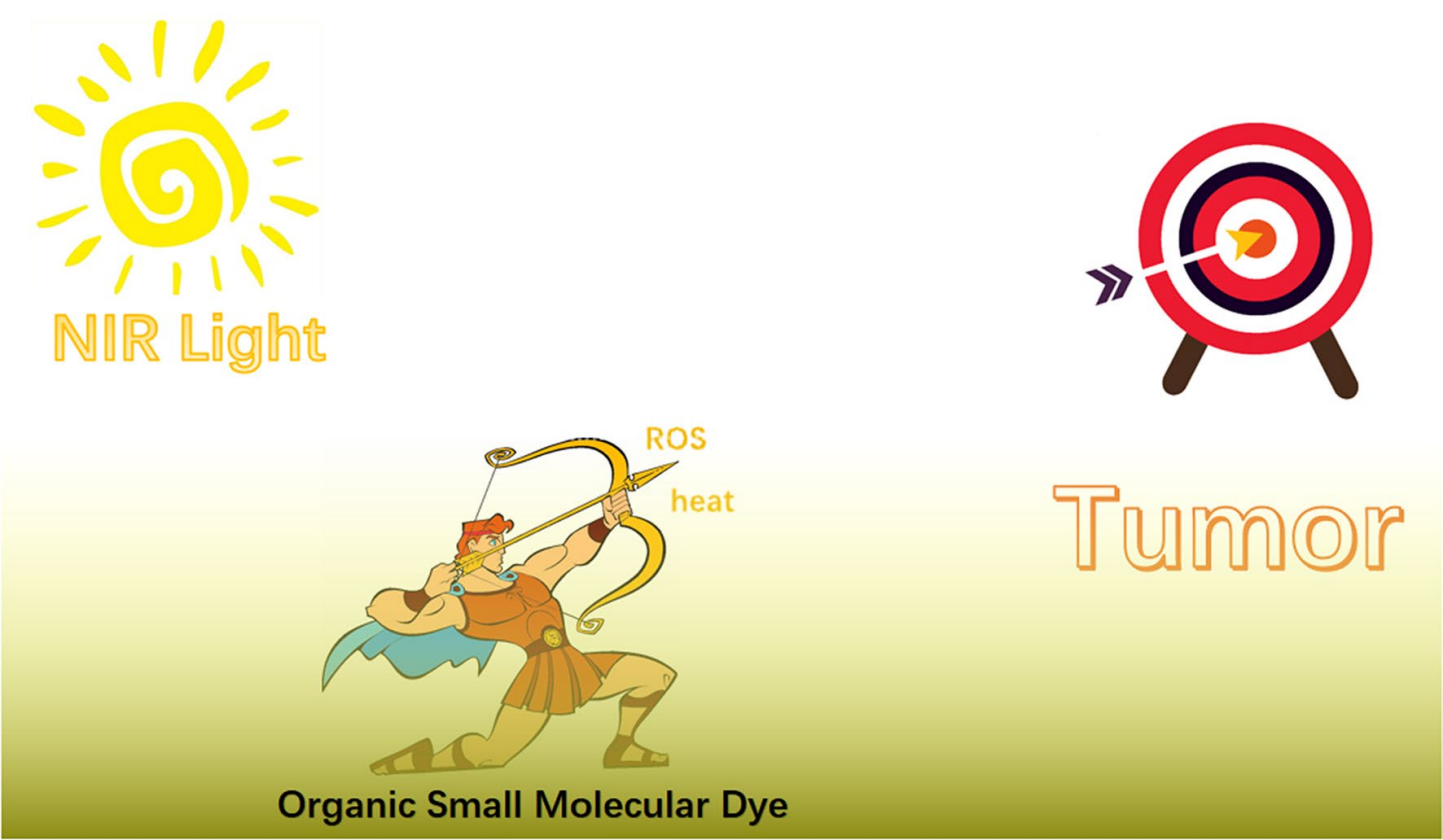

## Introduction

Cancer is an aggressive disease that causes cells to grow and divide. Given that cancer is a disease with high morbidity and mortality, and an incompletely elucidated mechanism of development and recurrence in humans, its treatment is facing great challenges. Traditional surgery, radiotherapy, and chemotherapy remain the most suitable clinical cancer treatment strategies. However, they have some limitations, such as serious side effects, multidrug resistance, limited efficacy, and poor effect on metastatic tumors [1]. The development of nanomedicine has ushered in a new field in cancer therapy that is expected to improve therapeutic efficacy and decrease side effects in cancer treatment [2].

Photothermal therapy (PTT) is a perspective minimally invasive method by using absorbents to generate heat under near-infrared (NIR) irradiation to destroy tumor at specific locations [3]. PTT is developing rapidly due to the good biosafety and low attenuation of NIR light in tissues. Moreover, it can overcome the shortcomings of nonselective tissue heating that are encountered in traditional hyperthermia and avoid serious side effects [4]. Traditional hyperthermia produces a maximum temperature gradient on the body surface, and most of the energy dissipates in healthy tissue in the external radiation path [5]. By contrast, PTT can concentrate the energy of NIR light on tumors to induce tumorigenesis [6]. Thus, local thermal destruction minimizes the adverse impacts on normal tissue [7]. Numerous photothermal agents have been extensively studied to enhance the photothermal conversion efficiency, overcome the disadvantage of inhomogeneous heat distribution, and thereby improve the thermal lethality of subcutaneous tumors. Some of these agents have been used for clinical research [1]. The nanomaterials with strong absorbance in the NIR region (700–1000 nm) are the most concerned. Currently developed photothermal agents mainly include noble-metal nanostructures, transition-metal chalcogenides, C-based materials, and organic materials [1].

Au nanostructures are the most widely used photothermal noble-metal materials. Up-converting rare-earth nanophosphors (UCNPs), Pd nanosheets, and Ag–Au alloys are used as photothermal materials in addition to Au [8]. Among transition metals, sulfides, such as Cu_x_S_y_, Cu_2_-xSe, MoS_2_, WS_2_, FeSe_2_, FeS, and TiS_2_, and oxides, such as MoO_x_, W_x_O_y_, and Ti_8_O_15_, are often used as photothermal agents [8]. C-based materials include single-walled C nanotubes (CNTs), graphene, graphene oxide (GO), and reduced GO [8]. The last type is organic materials, such as polymers of polyaniline (PANI), polypyrrole, and poly(3,4-ethylenedioxythiophene)-poly(4-styrenesulfonate), and the organic dyes loaded with protein-based nanocomposites, micelles or liposomes [9]. Organic small molecule dyes (OSMDs) are organic photothermal materials that can be used as photothermal agents and have the characteristics of low toxicity and good biocompatibility [10]. Furthermore, they can be immobilized onto metal or nonmetal materials. This approach improves OSMDs stability greatly to further exploit the photothermal conversion effect and reactive oxygen species (ROS) generation of OSMDs [11]. Nevertheless, the photothermal transfer efficiency of OSMDs is lower than that of metal agents. However, under NIR light irradiation, OSMDs can not only generate heat through the PTT effect but also ROS through the photodynamic therapy (PDT) effect by acting as a photosensitizer (PS) [12]. PTT combined with ROS therapy can achieve considerable results by working together to destroy cancer cells and improve the therapeutic effect.

ROS, which refer to radical or non-radical molecules derived from O_2_, as well as singlet O (^1^O_2_), peroxide (O_2_^2−^), superoxide (O_2_^**∙**−^), and the hydroxyl radical (HO^**∙**^) [13], are produced during photosynthesis in chloroplasts and aerobic respiration in mitochondria. They are also formed in the cytoplasm and peroxisome [14]. ROS play an important role in the redox regulation of ion channels, transcription factors and protein phosphorylation [15]. At stable concentrations, ROS can be used as messengers to regulate physiological processes. However, excessive ROS will produce toxic effects leading to cell death and tissue dysfunction [16]. This effect inspired scientists to use ROS to destroy cancer cells. Many compounds, such as arsenic oxide, Pt-based drugs, paclitaxel, and doxorubicin, play anticancer role by promoting the production of cellular ROS [13]. The use of ROS-induced toxicity for cancer treatment is also the research focus in the anticancer field. A highly effective platform based on PDT has been gradually established and developed. However, PTT cannot eliminate all tumor cells. Because the distribution of heat generated is uneven, especially in areas close to large blood vessels, heat can be quickly dissipated by circulating blood, leading to insufficient dose in this area. As far as PDT is concerned, its mechanism is that oxygen in the tissue generates ROS through interaction with PSs. However, the oxygen content in tumor tissue is highly heterogeneous. Due to the heterogenous distribution of blood vessels, severe hypoxia may occur in areas where tumor cells may produce drug resistance, thereby reducing the efficacy of PDT. In contrast, OSMD combines the heat generated by PTT and ROS generated by PDT under NIR irradiation, exerting synergistic anti-tumor effect. Therefore, the construction of an OSMD nanoplatform can not only improve the photothermal efficiency of dyes and the production rate of ROS but can also improve the stability of dyes in vivo. At the same time, the high biotoxicity and biocompatibility to the human body of OSMDs provide new opportunities for future cancer treatment.

## Near-infrared light

With the development of light generation, transmission and sensing technology, the application of photon technology in the medical field has made great progress [17]. Different kinds of light are used in different treatments, and lighting conditions can affect treatment. For example, in imaging diagnosis, light invasion should be minimized to avoid tissue damage. Meanwhile, tumor resection surgery requires laser energy to remove target lesions [18]. In short, both approaches are associated with the interaction between light and tissue [19]. In the process of light penetrating tissues, the reflection, absorption, transmission, and scattering may also occur as in the external environment [19]. The thermal effect or catalytic effect caused by light irradiation may change the enzyme activity, thus affecting the biological activity. Therefore, the interaction between light and tissue is crucial for the use of light in clinic [18].

The interaction between light and pathological tissue are decided by the optical properties of the pathological tissue and the light wavelength [19]. Although changing the nature of the pathological tissue is very difficult, this situation can be circumvented by changing the wavelength of light. Light irradiation is often used in surgery to ablate lesion. However, the thermal effect may damage normal tissue [19]. X-rays, ultraviolet, and NIR, which are three common types of light with specific wavelengths, are powerful tools for analyzing the interactions between light and tissues. In contrast to X-ray and ultraviolet, NIR provides noninvasive, spatiotemporal precision adjustment on demand [20]. Moreover, NIR light is quite safe, because compared with other wavelengths, the absorption of the major tissue chromophores (hemoglobin, myoglobin, and melanin) in the NIR range is relatively low, therefore, its tissue penetration is low [21]. Therefore, NIR light has more potential applications in tumor diagnosis and treatment than other wavelengths of light.

As the biological window [22], NIR has the characteristics of low absorption and strong penetration [23]. Compared with NIR-I window (650–900 nm), the NIR-II window (1000–1700 nm) has higher imaging quality and stronger penetration to tissues [24]. Relevant research has shown that NIR light penetrates tumor tissues more than normal tissues. For instance, NIR light with the wavelength of 630 nm penetrates normal brain tissue by approximately 0.9 mm and lung carcinoma by approximately 1.6 mm. As can be inferred from this information, NIR has selectivity for tumor cells [25]. In addition, 0.33 W/cm^2^ is the safe density limit for the 808 nm and 0.726 W/cm^2^ for the 980 nm, which are the common wavelengths of NIR in PTT [26, 27]. Many materials can produce thermotherapy effects under NIR irradiation. The phenomenon of light conversion to thermal power is called the photothermal effect.

In recent years, NIR has been widely used in induction imaging and selective therapy because of its strong tissue penetration and low tissue damage. PTT [28] and PDT [29] are two current methods for NIR-induced cancer therapies, which kill tumor cells by heating or producing ROS. NIR light can also transform heat-conducting nanoparticles (NPs) through photothermal effect, thus controlling the nanosystem of drug delivery [30]. Photothermal agents are the major basis for the application of NIR light. Various materials, especially OSMDs, have their own advantages and disadvantages in NIR PTT.

## Main types of organic small molecule dyes

**Fig. 1:**
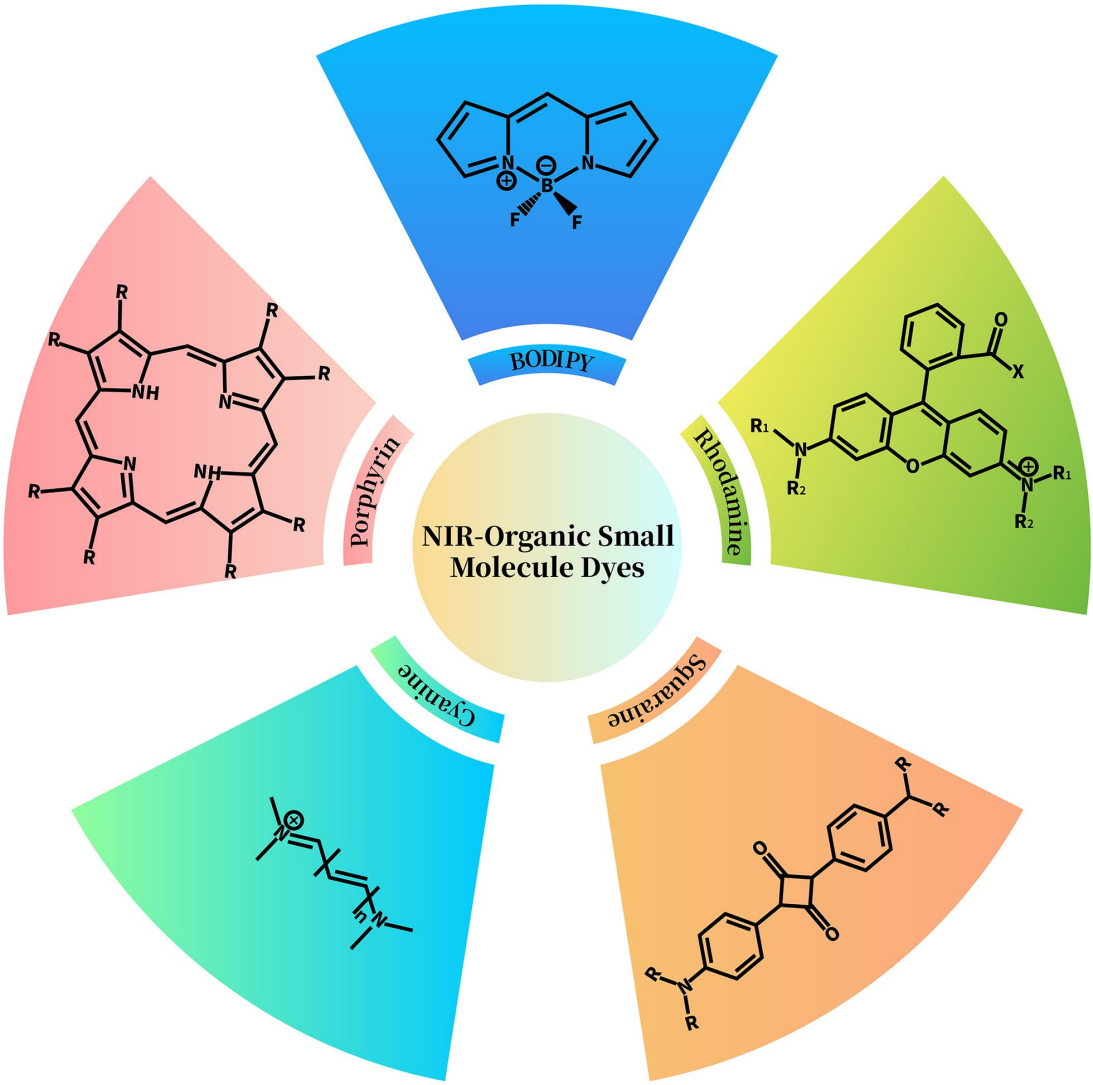
The structures of NIR-organic small molecular dyes

In addition to the necessary light source, photothermal transduction agents (PTAs) are indispensable for PTT. PTT depends on the heat generated by tumor cells exposed to NIR light and the photo-thermal effect of PTAs [31]. The light absorbed by photoactive molecules decays mainly through three relaxation pathways, including non-radiative transition, radiative transition, and inter-system crossing, which compete with each other. Among them, the non-radiative transition generates heat for PTT. Due to the total amount of decay of fixed active molecules, it is necessary to suppress the other two pathways in order to improve the PTT effect. In a tightly packed molecular system, photoexcited molecules undergo energy transfer and/or intramolecular electron transfer, which can stimulate intrinsic fluorescence and significantly improve photothermal conversion [32]. The assembly or aggregation of photoactive molecules into nanomaterials not only improves photothermal properties, but also enhances drug accumulation at tumor sites through enhanced permeability and retention (EPR) effects. PTAs are classified into inorganic nanomaterials, OSMDs, and polymeric agents. Among them, the inorganic nanomaterials are usually metal nano-ions, and exhibit good NIR absorption properties and high photothermal efficiency through their surface plasmon resonance. Polymer-based PTAs achieve NIR absorption and photothermal conversion through their highly conjugated structures. On the other hand, OSMDs-based PTAs can be simply chemically modified to reach the desired wavelength and achieve specific targetability. PTAs can absorb and transform light energy into heat, thus increasing temperature to kill the tumor cells [31]. Under the guidance of various imaging methods, the efficacy of PTT based on OSMDs has shown extraordinary results [12]. NIR-OSMDs have become an important part of many biomedical applications, especially PTT (Table 1) [33]. Many structural types of NIR-OSMDs with unique properties exist (Fig. 1). There are several strategies to achieve high photothermal conversion efficiencies (PCE) for OSMDs, including modulation of intramolecular rotation or intermolecular interactions, wavelength extension from NIR-I to NIR-II windows, self-assembly strategies, nanocarrier-assisted strategies, and stimulated activation strategies. These strategies include modulation of single molecule properties and synergistic improvement of molecular properties [34].

**Table 1:** The OSMD-based nanoplatforms for NIR photodynamic and photothermal co-therapy

Therapeutic method	Nanoplatform		Size	NIR wavelength	Application	References
PDT/PTT combination therapy	Cyanine	AuNR/ICG vesicle	238.46 nm	785 nm	PC3 cells	[125]
		ICG-PEI-HAuNs	122.50 nm	880 nm	SKOV3 cells	[126]
		AuNR@MSN-ICG-RLA/CS(DMA)-PEG	78.50 × 37.20 nm	808 nm	MCF-7 cells	[127]
		TNYL-ICG-HAuNS	70 nm	880 nm	SKOV3 cells	[135]
		FAL-ICG-HAuNS	151 nm	890 nm	CT-26 cells	[136]
		Fe_3_O_4_@PDA@CaCO_3_/ICG	17.40 nm	700–900 nm	4T1 cells	[139]
		Fe-PDAP/GOx/ICG	48.90 nm	720–786 nm	MDA-MB-231 cells	[140]
		Cu_9_S_5_@mSiO_2_-ICG@PEG-LA	50 nm	808 nm	HepG2 cells	[141]
		HA-Ti@GO/ICG	–	700–900 nm	4T1 cells	[142]
		NGO-808	20–40 nm	808 nm	A549, Lewis tumor cells	[149]
		ICG/rPAA@SWCNTs	420 nm	808 nm	Hela cells	[158]
		COF@probe	–	808 nm	3T3 cells	[159]
		COF@ICG@OVA	100 nm	650 nm, 808 nm	H22 cells	[160]
		UCNPs@PDA-ICG	40 nm	808 nm	H22 cells	[161]
		HA-PB/ICG	290 nm	808 nm	4T1 cells	[163]
		HA-PEG-CyI	112.50 nm	808 nm	4T1 cells	[164]
		M@PPI-siRNA	190.50 nm	808 nm	SCC-25, B16 cells	[165]
		iRGD-rHDL/ICG	86.70 nm	780–800 nm	4T1 cells	[167]
	Porpyrin	P-4	46 nm	714 nm	Hela cells	[170]
		AuNR@SiO2-TCPP	190 nm	660 nm, 808 nm	A549 cells	[175]
		PF6-Au	60–100 nm	400–800 nm	A549 cells	[177]
		Pp4N/GNR	50 nm	660 nm, 808 nm	3T3-L1, 293 T cells	[201]
		GO-C_60_	100–400 nm	650, 808 nm	Hela cells	[202]
		VONc-COF-Por	140 nm	808 nm	MCF-7	[204]
		TPAPor	80 nm	690 nm	Hela cells	[209]
		PB@PCN@MEM	110–140 nm	660, 808 nm	CT26 cells	[210]
	Rhodamine	PSBTBT-Ce6@Rhod	149 nm	675 nm	MCF-7 cells	[215]
	Squarine	BSA-Cy7-SQ/GM	300 nm	808 nm	HepG2, HL7702 cells	[219]
	BODIPY	BDPTPA	40–160 nm	808 nm	143B cells	[223]
		FMAB NPs	68.10 nm	775 nm	Hela cells	[225]
		MTAB NPs	155 nm	808 nm	4T1 cells	[226]
Imaging-guided phototherapy	Photoacoustic imaging	COF-366	100 nm	635 nm	4T1 cells	[238]
	Fluorescence imaging	Cypate, Cy7, CyI	–	804 nm, 776 nm, 750 nm	HepG2 cells	[244]
		AgSiO_2_-PpIX	125 nm	630 nm, 698 nm	–	[245]
		AuNRs@SiO_2_-IR795	19.40 nm-82.50 nm	795 nm, 812 nm	U251MG, HepG2 cells	[247]
	Magnetic resonance imaging	FeS_2_@C-ICG-PEG	200 nm	808 nm	HepG2 cells	[253]
		GNRs@BPP-Gd	80 nm	808 nm	4T1 cells	[256]
	Multimodal imaging	ICG-PtMGs@HGd	92.50 nm	800 nm	4T1 cells	[262]
		HSA-ICG	75 nm	671 nm	4T1 cells	[263]
		ICG-Ag@PANI	115.70 nm	808 nm	Hela cells	[264]
		PMATIB/PEI/Au nanoshell/HA	187 nm	808 nm	MCF-7 cells	[265]
		FCCP NPs	100 nm	635 nm, 980 nm	A549 cells	[271]
Synergistic chemotherapy and phototherapy	Synergistic chemo/PDT/PTT	cRGD@TAT-DINPs	51.18 nm	808 nm	MDA-MB-231, A549 cells	[300]
		DOX/ICG@biotin-PEG-AuNC-PCM	109.10 nm	800 nm	MCF-7 cells	[301]
	Imaging-assisted tri-modal therapy	DZSM NPs	296.90 nm	638 nm, 808 nm	4T1 cells	[275]
		HMPB@PEI/ICG/DOX	310.10 nm	808 nm	4T1 cells	[298]

### Cyanine dyes

The cyanine dye has a positive charge and two nitrogen centers (Fig. 1). This polymethine structure is the chromophore of cyanine [35]. As the simplest cyanine dyes, monomethine and trimethine cyanine (Cy3) exhibit an absorption wavelength in the visible region. Their absorption wavelength can be easily bathochromically shifted by approximately 100 nm by simply inserting one vinylene moiety into the linker bridge. Therefore, pentamethylcyanine (Cy5) has a high absorption wavelength that reaches the NIR spectrum (> 700 nm), and the absorption wavelength of heptamethinecyanines (Cy7) may exceed 1000 nm [36]. Furthermore, other functional groups on cyanine dyes can affect absorption wavelength [37]. For example, if the fused benzo ring is introduced into dihydroindole, it will cause a bathochromic shift of 20–30 nm [36]. Since their discovery, cyanine dyes have found numerous applications in various fields, including nonlinear optical materials, organic photovoltaics, chemical sensors, and fluorescent probes [11]. In recent years, cyanine dyes, especially indocyanine green (ICG) [38–40], which has been approved by FDA for almost over 50 years, are very popular in PTT field [41]. The main ways to enhance the PCE of cyanine dyes are currently J-aggregates [42], wavelength extension from NIR-I to NIR-II windows [43], and self-assembly strategies [44].

### Boron dipyrrin dyes

Boron dipyrrin (BODIPY) dyes feature a chemical structure wherein two pyrrole rings are linked by a methine in a tricyclic fused ring and a six-membered ring with a boron atom flanked by two five-membered rings (Fig. 1) [11]. The dye has the advantages of high fluorescence quantum yield, high extinction coefficients, good photostability, pH insensitivity and so on, which makes it more applicable [45–48]. The absorption wavelength of BODIPY dyes is usually in the visible region, and it can also produce fluorescence between 470 and 550 nm, which are not in the NIR region. Therefore, a large number of studies have focused on obtaining NIR-BODIPY dyes by modifying their basic structure [45]. Substituents, such as aryl, alkynyl, and styryl, can effectively extend the absorption wavelength of BODIPY dyes from 660 to 730 nm and even to 808 nm for deepened tissue penetration [11]. Moreover, due to the proton acceptor nature of diethylamino groups, BODIPY dyes exhibit pH-triggered enhanced PTT–PDT efficacy and thus show great potential for application in phototheranostics [49]. The main ways to enhance the PCE of BODIPY dyes are intramolecular rotation [50], self-assembly strategy [51], J-aggregates [52], and wavelength extension from NIR-I to NIR-II windows [53].

### Rhodamine dyes

Rhodamine dyes, together with fluorescein and eosin dyes, belong to the family of xanthenes (Fig. 1) [54], which are very popular and widely used due to their large molar absorbability, photobleaching resistance and high quantum yields. The absorption/emission wavelengths of classical rhodamine dyes, such as rhodamine 101, rhodamine B, and rhodamine 6G, are in the visible range [54], which is not conducive to fluorescent imaging and phototherapy. Scientists have thought of many ways to obtain NIR rhodamine dyes. These approaches include thickening the aromatic heterocyclic ring to enlarge the xoxanthracene ring [55]; introducing strong electron-withdrawing groups, such as –CN [56]; and replacing O-bridge atoms with other atoms, such as N and S [57]. However, the NIR rhodamine compounds prepared through the above methods have complex synthesis processes, low absorption and fluorescence intensity, and small Stokes displacement (< 35 nm) [58]. Recently, a number of novel NIR rhodamine compounds that are relatively simple to synthesize and possess high quantum efficiency and great potential in biological imaging and tumor therapy have been reported. They include Si-paironin, Group14 rhodamine [59], and Changsha near-fluorescent dye [58].

### Squarine dyes

Squaraine (SQ) dyes are usually synthesized through condensation reactions between square acid derivatives, methylene bases, and electron-rich aromatics (Fig. 1). They have strong absorption and emission in the NIR region because of their large planar π-conjugated system and donor–receptor–donor structure that easily forms a stable amphoteric rigid plane resonance structure [60]. SQ dyes have good physical and chemical properties, high absorption peaks, strong NIR emissions, high molar extinction coefficients, and good photoconductivity [61]. Considerable effort has been done to obtain SQ dyes with high hydrophilicity and NIR absorption. For example, KSQ-4-H, a SQ dye synthesized by attaching four water-soluble sulfonic acid groups to the main chain of SQ, can be completely dissolved in phosphate-buffered saline and has an obvious absorption peak at 775 nm [62]. Two SQs were conjugated by thiophene or pyrene units to produce bivalent acid dyes that readily bind to bovine serum albumin (BSA) as noncovalently labeled probes and exhibit enhanced fluorescence in the NIR region [63]. J-aggregate SQ NPs were obtained by packaging the fluorescent dye bispyrrole-sq-bispyrrole into the amphiphilic copolymer F-127 through the J-aggregation method (sliding stack arrangement). The NPs had good fluorescence performance and PTT photothermal conversion rate [64]. The main ways to increase the PCE of squaraine dyes include J-aggregation [64] and wavelength extension from NIR-I to NIR-II windows [65].

### Porphyrin dyes

Porphyrin (Por) is a macromolecular heterocycle formed by interconnection of four pyrroles through four methine bridges, which is a highly conjugate system. This structure can produce strong ring current, as shown in Fig. 1. This kind of dye has strong Soret band (approximately 400 nm) and Q-bands (500–600 nm) in the visible region [66]. Its absorption spectrum is easily redshifted to the NIR region by coupling different aromatic rings, including aromatic hydrocarbons or aromatic heterocycles, around the Por ring to expand its π-conjugation system. Therefore, different aromatic rings are fused in the periphery of the Por ring, and the main modes can be classified into β,β-arene-fused Pors; meso-β-arene-fused Pors; and β-meso-β-arene-fused Pors [66]. Furthermore, N-confused Por (NCP) can be obtained by inverting a pyrrole ring into a normal Por ring and further connecting groups, such as an alkyne group, to the central sp_2_ hybrid C atom to obtain vinylidene bridged NCP. The interaction of functional groups in the rings can expand the π-conjugate system of the Por rings, and the HOMO energy can be significantly increased such that the maximum absorption wavelength of the Por rings in CH_2_Cl_2_ is 775 nm [67]. The center of macrocyclic Pors can be modified by replacing some N atoms in the pyrrole ring with S or Se atoms. This approach can increase the intracavitary intermolecular H bonding of the modified Por relative to that of all Aza-type macrocyclic Pors. The absorption and fluorescence wavelength of the modified Por are redshift because of the increased planarity of its molecular structure [68]. The photothermal conversion of porphyrin dyes can be greatly improved by self-assembly strategy [69].

The ideal fluorescent dye should have a NIR absorption/emission peak, large Stokes shift, stable optical properties, good water solubility, low biotoxicity, and certain reactive functional groups for high performance and multifunctional modification. Through the efforts of scientists, many NIR-fluorescent dyes with excellent performance have been obtained in recent years [70].

## Photodynamic therapy

As a result of an imperfect vascular system, solid tumors usually proliferate rapidly and exceed their blood supply, leaving areas in a hypoxic microenvironment [71]. This situation leads to the ineffectiveness of various cancer treatments, such as chemotherapy, radiation therapy, and hyperthermia [72]. Tumor hypoxic areas may not be treated, leading to tumor metastasis or recurrence [73]. The subsequent development of hyperbaric oxygen (HBO) therapy, which delivers a high concentration of O to the body and tumor through pressurization, provides a solution to this problem [74]. Clinical results showed that despite the recurrence rate of more than 50%, tumors disappeared completely in 44% of the patients with malignant glioma treated with radiotherapy combined with HBO [75], indicating that the strategy of increasing the O content in tumor hypoxic areas is feasible. This situation has been further improved with the development of PDT.

**Fig. 2:**
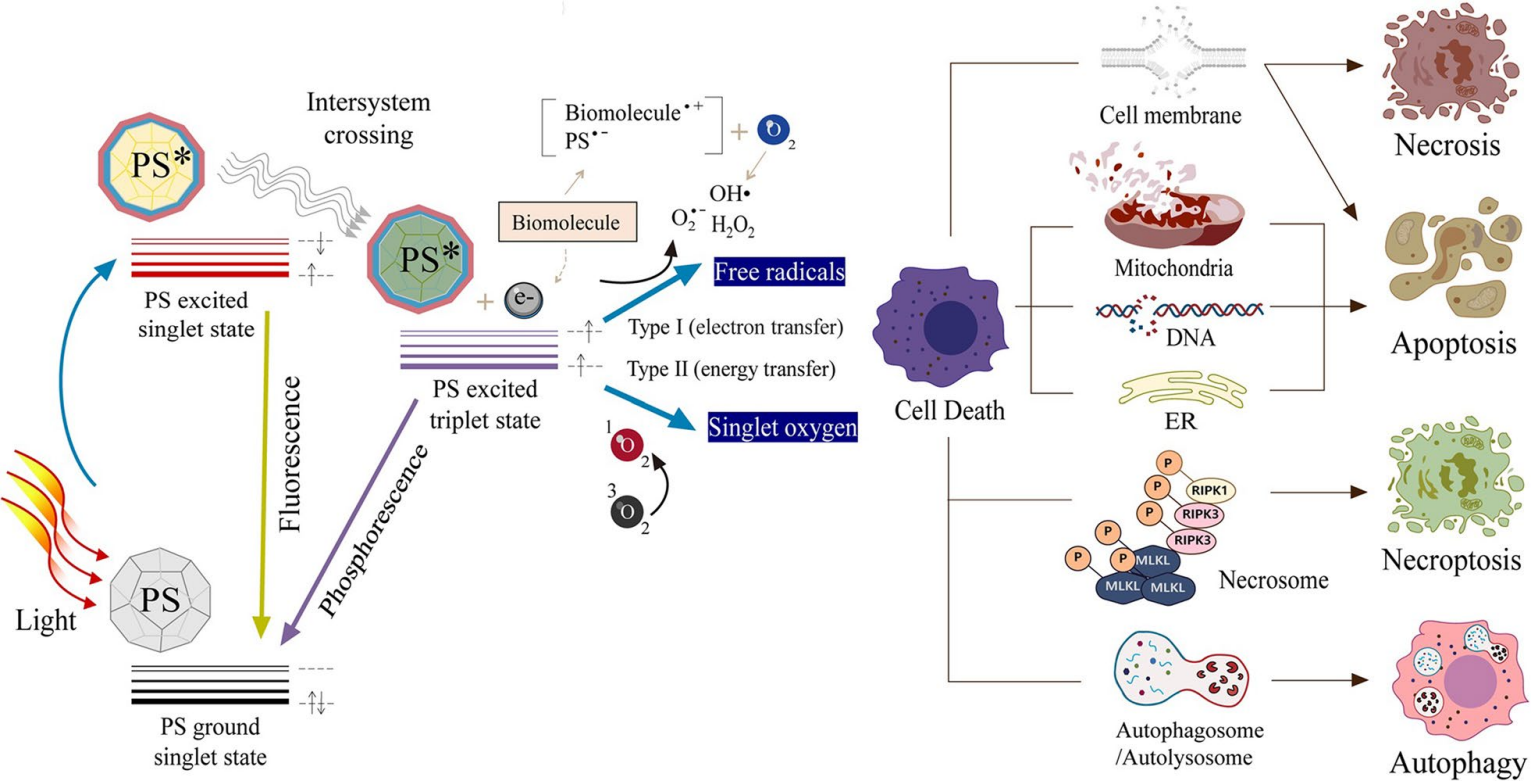
Schematic illustration of photodynamic reactions (either type I or type II) and cell death pathways in the process of PDT. A PS absorbs energy from light to kill tumor cells via ROS generation. The PDT-induced modes of cell death, including apoptosis, necrosis, necroptosis, and autophagy, depend on the cell type, PS type or concentration, intracellular localization, light dose, and oxygen partial pressure [77]

PDT, which uses exogenous ROS produced by photoactivating PSs to eliminate tumor cells, has become a promising treatment for cancer and microbial infections [76]. The basic elements of PDT include three kinds: specific light sources, photosensitizers (PSs) and oxygen-containing substrates (e.g., O_2_ and water) [13]. The photodynamic procedure can be divided into type I or type II process (Fig. 2) [77, 78]. The type I process generates free radicals through direct activation reaction of electron or H atom transfer. In contrast to those in the type I process, in the type II process, the electronically excited PSs dominated by most dyes react with O to form ROS [13].

### Selection of photosensitizers

PS is a key component of PDT, which is responsible for generating ROS at the corresponding wavelength (Fig. 2) [79–81]. When it is distributed in the plasma membrane, PDT will cause necrosis of the cells [82]. However, when it is located in other organelles, such as the plasma membrane, nucleus, mitochondria, endoplasmic reticulum, and lysosomes, PDT oxidative stress can also induce apoptosis [83–87]. Furthermore, PDT leads to necroptosis by forming necrosomes involving receptor-interacting protein kinases 1 and 3 [88, 89]. Autophagy is also a way of cell death through photosensitization [90, 91]. Generally, PSs are usually pure and stable compounds that have high cross-efficiency from the singlet to the ternary system, very low dark toxicity, minimal phototoxicity to normal tissue damage, and maximum absorption at long wavelengths [92]. At present, many PSs, which can be divided into inorganic PSs and organic PSs, have been used in PDT.

#### Inorganic materials for PDT

In recent years, material synthesis technology is developing faster and faster, inorganic materials with different elements and different functional properties are expected to be applied in biology and medicine, including PSs for PDT [1]. Cu ions, a common inorganic metal material, are often used as PSs. The integration of Cu^2+^ and photosensitive G–C_3_N_4_ nanosheets (Cu^2+^–G–C_3_N_4_) enhance the production of phototriggered ROS and the depletion of intracellular glutathione levels. Reduced glutathione can decrease the consumption of ROS produced by light exposure to improve efficiency. The redox active Cu^2+^–G–C_3_N_4_ can catalyze the reduction of O_2_ into O_2_^**∙**−^ anions or H_2_O_2_ into HO^**∙**^ and promote the generation of ROS [93]. The synergistic effect of improved ROS production and glutathione consumption could improve the effectiveness of PDT in cancer treatment. In addition, the high reactivity of MnO_2_ NPs loaded onto multifunctionalized chlorine E_6_ (Ce_6_) modified by polyethylene glycol (PEG) was used to promote the endogenous H_2_O_2_ to generate O_2_ in the tumor microenvironment to enhance tumor-specific PDT [94]. Moreover, UCNPs are highly representative inorganic materials for PDT. They are not only used as carriers, but also as energy donors of PS through the influence of fluorescence resonance energy transfer upon NIR light irradiation [95].

#### Organic materials for PDT

Since a hemoporphyrin derivative was first used to inhibit tumor growth in mouse mammary fat pads in 1975, Pors and their analogues have been used in PDT successively and are thus regarded as the first generation of organic PSs [2]. However, Por-based PSs have limited applications in PDT due to their poor water solubility, low clearance rate, and lack of long wavelength absorption [96]. The second generation of organic PSs was gradually developed to overcome the shortcomings of Por-based PSs. They are chemically synthesized pure compounds, such as phthalocyanines, chlorines, and some biological dyes, with the characteristics of constant composition, strong long-wavelength absorption in the 650–800 nm range, optimal tissue penetration, fast tissue clearance, and high red absorption extinction coefficients [97]. Various inorganic/organic nanocarriers, such as liposomes, micelles, dendritic macromolecules, and mesoporous silica, were developed as third-generation materials for the delivery of PSs with enhanced targetability and stability [2]. Furthermore, on the basis of available PSs, some functional organic groups, such as PEG [98], triphenylamine [99], the polypyridyl ruthenium complex [100], and C chain liposomes [101], can be introduced to obtain special surface activities, increase ^1^O_2_ production efficiency, and improve cytophagy and targetability for tumor cells [1]. For example, protoporphyrin IX (PpIX) in PS nanocapsules was prepared by using PEG-modified dendrimers. PpIX in the nanocapsules exhibited stability under physiological conditions during PDT application. In addition, the stability of adsorption performance can be enhanced by exploiting the internal hydrophobicity of PEG-poly(propylene imine) (PPI). Given its high level of ^1^O_2_ production and efficient delivery to mitochondria, the complex of PpIX with PEG–PPI exhibited more efficient cytotoxicity than free PpIX [98]. Through combined application with PEG, the selective photoinduced modulation of DNA can be introduced into the application of PSs for the spatial and temporal control of the photochemical reaction in phototherapy. For example, the photoreactive peptide nucleic acid (PNA) can be used to conjugate the PS Rose Bengal (RB) with PNA–RB, which exhibits sequence specificity, cell permeability, and photoactivation in the visible region. PNA–RB conjugates with synthetic DNA show enhanced photoactivity and support the involvement of ^1^O_2_ and may provide useful approach for selective PDT [102]. Moreover, the encapsulation of PSs into PEG is conducive for enhancing phototoxicity. *m*-Tetra(hydroxyphenyl)chloride (*m*-THPC) was encapsulated as a PS into folic acid (FA)-targeted PEG liposomes to prepare a new PDT drug delivery system [103]. FA-targeted PEG liposomes increased the uptake of the embedded *m*-THPC by twofold and enhanced the photoinduced cytotoxicity of KB cells with high levels of folate receptors by 1.5-fold due to photoinduced ROS production. FA-modified liposomes have good targeting function [104] and can also be used to target ligand-functionalized hollow mesoporous silica NPs (HMSNPs) for loading 5-aminolevulinic acid (5-ALA), a precursor of PpIX, to destroy skin cancer cells through PDT [105]. A targeting ligand makes 5-ALA-loaded HMSNPs selectively internalize into cancer cells. PpIX formed through 5-ALA release showed high phototoxicity in vitro. In addition, some hormones are specific for cancer PDT [106].

Organic PSs have greatly promoted the development of PDT due to their high ^1^O_2_, good biocompatibility, and structural changes. OSMDs, a member of the family of organic PSs, have a good NIR range and ^1^O_2_ production rate and little toxic and side effects. They also have imaging functions and a PTT effect that provide great help and many new ideas for enriching PDT [107].

### Application of organic small molecular dyes in PDT

Given their merits of clear chemical structure, good reproducibility, and excellent biocompatibility, OSMDs are more promising as PSs for PDT compared with their inorganic and polymeric counterparts [11]. Many examples of BODIPY dye modification to improve the effectiveness of PDT have been reported. For example, one work focused on a bromo-substituted BODIPY with high light stability (I) composed of thiopyrrole. The results of MTT method and flow cytometry showed that this material had a high single-state O quantum yield and phototoxicity in the NIR region after irradiation and could be used for photodynamic analysis [108]. Another study reported a compound based on a two-stage PS trap molecule, in which the PS and the trap consisted of BODIPY and the mycophenol ring of α-tocopherol, the most potent natural antioxidant. The photodynamic inactivation of ROS in Gram-negative *Escherichia coli* successfully demonstrated the effectiveness of this selective photoactivated ROS in generating ^1^O_2_ in cells [109].

In addition to BODIPY, Pors have many applications in PDT. A nanoplatform (PFL-AuC) was constructed by using a PpIX, FA, and NIR-emitting Au cluster capped with lipoic acid for PDT. The triplet quantum yield of PFL–AuC has been greatly increased by 80% relative to that of PpIX alone (63%). More importantly, PFL–AuC containing 60 μg (0.136 mM) of PpIX was sufficient to destroy 50% of tumor cells. Histopathology analysis and fluorescence (FL) imaging demonstrated that PFL–AUC had an obvious and effective destructive effect on tumor cells, confirming the efficacy of PDT with PPFL–AUC in vivo [110]. Four new sensors containing the curcumin structural units were prepared by using 2-(2-hydroxy-naphthalene)-5,10,15,20-tetraphenyl Por and its Cu(II), Ni(II), and Zn(II) complexes as raw materials. Among these substances, Pors and curcumin were linked by 1,6-dibromohexane and possess antitumor activity. The cleavage ability of the compounds for pBR322 plasmid DNA under light and dark irradiation was detected through gel electrophoresis. The results showed that the new Por compounds had obvious photosensitive shearing activity and can effectively combine with DNA and were thus potential PSs [111]. The application of phthalocyanine compounds, a member of OSMDs, has also been extensively studied. Phthalocyanines derivatives conjugated with functional groups, such as octacarboxyl gallium, amino-terminal fragments, heavy atoms (Br and I), liposomes, glycerol, gonadotropin-releasing hormones, phosphonic acid, erlotinib, and *o*-carborane have been synthesized [1]. For example, erotinib–Zn(II) phthalocyanine can produce ^1^O_2_ under light to kill human hepatoma (HepG2) cancer cells with a very low light dose and the IC_50_ of between 9.61 and 91.77 nM. These results indicated that erlotinib–Zn(II) phthalocyanine is a promising PDT molecular targeting antitumor drug [112].

## PDT/PTT combination therapy of NIR-OSMDs

NIR-OSMDs have recently become an effective photothermal agent for PTT due to its biodegradability, low cost and toxicity, and strong NIR absorption over a wide wavelength range [113]. Nevertheless, PTT based on OSMDs has limited applicability due to its low photothermal conversion efficiency and inadequate destruction of the tumor areas that are unirradiated by NIR light. However, OSMDs can be used as photosensitizers to generate ROS to further eliminate cancer cells and extend the application range (Table 1).

### Cyanine dyes

Cyanine dyes have been used widely as traditional photothermal agents and PSs in PTT–PDT combination therapy. The combination of dyes and nanomaterials often produce unexpected results.

#### Combination with metal materials

Cyanine dyes combined with metals are widely used in PTT–PDT [114–118]. In particular, Au NPs are general materials in cancer treatment, which have strong absorption in visible area, low toxicity and good biocompatibility [119–123]. The most common type of cyanine dye is conjugating ICG [124]. Many exquisite designs for greatly improving the therapeutic effect of PTT–PDT exist. For example, through the coordination of cetyltrimethylammonium bromide with ICG and polycaprolactone on the surface of hydrophobic Au nanorods (AuNRs), biomimetic cell membrane polymer vesicles were generated to realize self-assembly in aqueous solution. The increase of vesicle stability makes PTT and PDT play a synergistic role in the treatment of prostate cancer [125].

However, many problems in the application of cyanine dyes remain. These problems include poor stability and targeting effect, and the photodynamic resistance of cancer cells. The stability of ICG is very important in the process of application. For solving the above problems, a nanoplatform (ICG–PEI–HAuNS) for PTT–PDT combination therapy was constructed, which used branched polyethylenimine to couple ICG to hollow Au nanospheres (HAuNS). This method increased the ICG load and kept the fluorescence and activity of ICG. Compared with free ICG or HAuNS, this material showed not only improved stability, photothermal conversion efficiency (from 22 up to 47.7 °C), and photodynamic effects, but also remarkably suppressed the growth of subcutaneous B16 tumors. Meanwhile, the synchronous effect of PDT and PTT under laser irradiation provided by ICG–PEI–HAuNS greatly reduced or even cleared the metastatic cells around liver, spleen, and lung microvessels [126].

In addition to enhancing the stability of ICG, antitumor therapy has focused on the targeting effect of nanomaterials. For example, AuNR@MSN–RLA/CS(DMA)–PEG was prepared to cross the biological barrier and realize the enhancement effect of PDT and PTT. ICG was loaded into AuNR@MSN first, and then the peptide RLA ([RLARLAR])_2_ with mitochondrial targeting and plasma membrane permeability was connected. Subsequently, the charge-reversible 2,3-dimethylmaleic anhydride (DMA)-modified chitosan (CS) oligosaccharide-block–PEG polymer was introduced to form AuNR@MSN–RLA/CS(DMA)–PEG with stealth properties [127]. Charge reversal was monitored by using zeta measurements at physiological environment (pH 7.4) and tumor acidic microenvironment (6.8), because charge reversal helped to successively cross biological barrier to improve tumor cell uptake and cycle time [128]. The zeta of AuNR@MSN-RLA/CS(DMA)–PEG increased significantly at pH 6.8 and plateaued in 3 h mainly because approximately 30% of the DMA amide bonds were converted into amino groups within 10 min upon incubation at pH 6.8 [129, 130]. However, because the DMA with negative charge was inert in the physiological environment [131], the zeta potential relatively slowly changed at pH 7.4 and remained negative even after 24 h of incubation. Therefore, under the condition of physiological pH 7.4, CS–PEG coupled with anionic DMA can mask the AuNR@MSN–RLA nanoplatform by electrostatic interaction. However, RLA peptide can increase mitochondrion targeting and cell uptake in acidic environment, which was also demonstrated by experiments [132]. Moreover, in the antitumor effect test, AuNR@MSN–ICG–RLA/CS(DMA)–PEG was the most stable and had the strongest tumor inhibition activity [127].

PTT–PDT based on OSMDs can also overcome the problem of drug resistance. The TNYL–ICG–HAuNS nanosystem was developed by covalently conjugating ICG and the TNYL peptide, which can interact with the EphB4 receptor to enhance tumor-targeting efficiency [133, 134], onto the surfaces of HAuNS to combine PDT and PTT. The nanosystem can significantly improve light stability, ROS generation rate, and PTT effect under NIR light, so it shows significantly enhanced anti-tumor activity. After repeated irradiation, TNYL–ICG–HAuNS could still generate ROS to reduce the expression of nuclear factor erythroid 2-related factor 2 (Nrf2), which causes PDT resistance [135]. An endoplasmic reticulum (ER)-targeting nanosystem consisting of ER-targeting pardaxin (FAL) peptides, ICG, HAuNs (FAL–ICG–HAuNS), and an O-delivering hemoglobin liposome for reversing hypoxia was further created based on the above effect. In contrast to nontargeting nanosystems, the created nanosystem induced robust ER stress and calreticulin, a marker for immunogenic cell death, under NIR light irradiation. Thus, antitumor efficiency was enhanced [136].

In addition to Au, Fe, Cu, and other metals can be used with cyanine dyes in PTT–PDT combination therapy [137, 138]. For example, based on photothermal properties of polydopamine (PDA) and the photodynamic properties of ICG, a low-toxicity nanocomposite composed of calcium carbonate and ICG-modified magnetic PDA NPs was developed for NIR-induced PTT–PDT therapy. The calcium carbonate layer can fix ICG on Fe_3_O_4_@PDA and release drugs in acidic environment. This nanocomposite can increase the cycle time of ICG significantly, thus improving the therapeutic effect [139]. An iron-doped polydiaminopyridine (Fe-PDAP) nanozyme loaded with glucose oxidase and ICG was used to react with H_2_O_2_ to produce O_2_, which can improve the treatment of oxygen-dependent PDT [140]. Furthermore, Cu_9_S_5_@mSiO_2_-NH_2_ was synthesized for the efficient loading of ICG to improve ROS generation and photothermal conversion efficiency. The combination of Cu_9_S_5_ and ICG significantly improved photothermal conversion efficiency (the temperature increased to 55.8 °C under 808 nm NIR light) and enhanced light-dependent ROS generation. Then, PEG and lactose acid (PEG–LA) externally grafted Cu_9_S_5_@mSiO_2_–ICG was prepared to further improve cell uptake and targeting function. Due to the synergistic effect of PTT and PDT, the cytotoxicity of Cu_9_S_5_@mSiO_2_–ICG@PEG–LA on HepG2 cells was increased [141]. Ti in combination with ICG also showed good performance in the treatment of PTT and PDT. Ti@GO grafted with hyaluronic acid (HA), which was used to improve water solubility and targetability, could significantly increase the stability and drug accumulation of the model drug ICG in cells, improve the efficiency of tumor phototherapy, and reduce light-related side effects [142]. Similarly, MnO_2_ and MoO_2_ had also been applied to reinforce the phototherapeutic efficiency of ICG [143, 144].

#### Combination with non-metallic materials

Nonmetallic materials can also be combined with OSMDs for PTT–PDT combination therapy [145], among which GO and CNTs are the most widely used materials [146, 147]. GO is a good drug carrier and has strong absorption in NIR. Therefore, it can be combined with PTT to treat tumors [148].

**Fig. 3:**
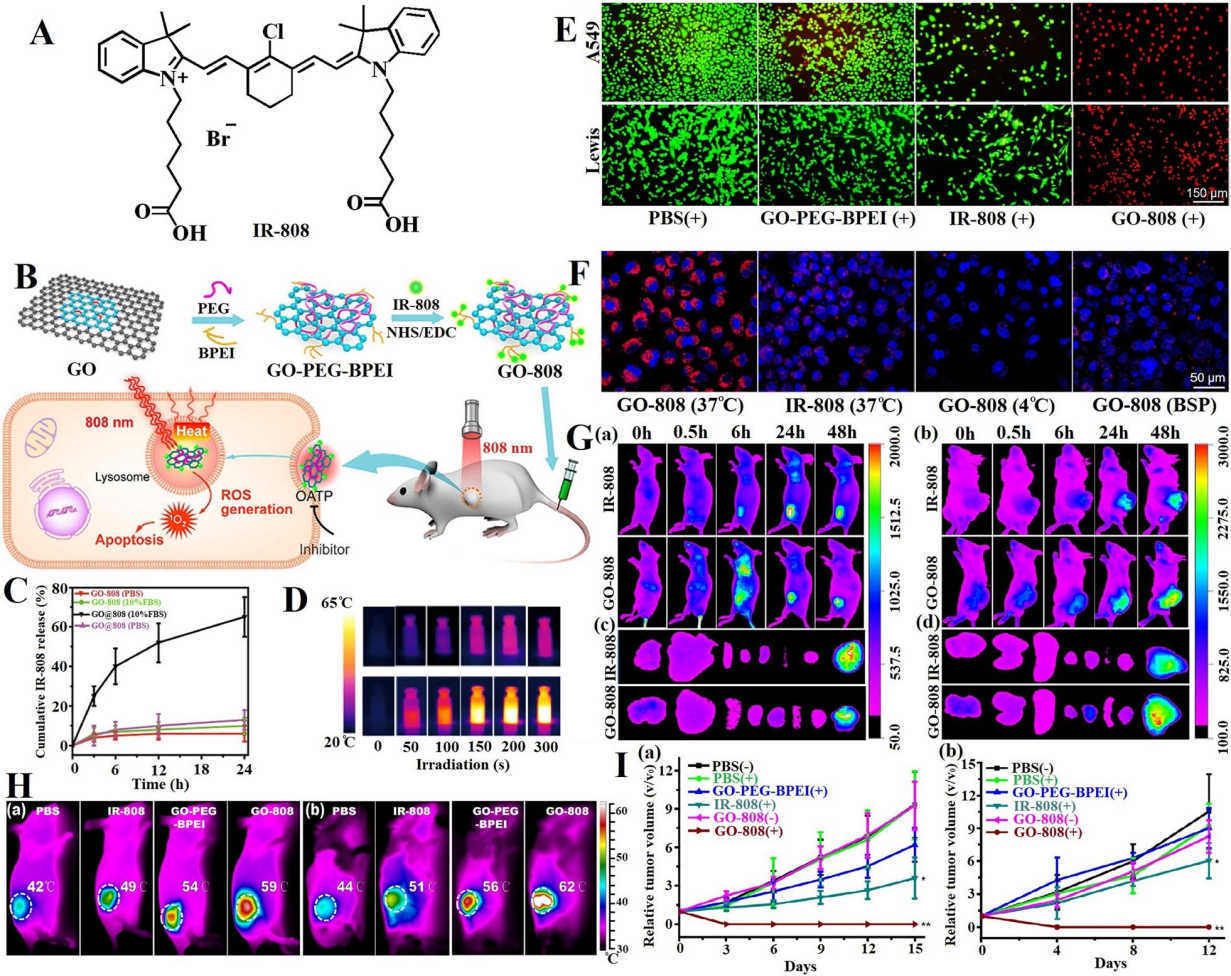
A multifunctional photosensitizer, heptamethine indocyanine dye, grafted on PEG and BPEI dual-functionalized GO for synergistic phototherapy. **A** Structure of heptamethine indocyanine dye. **B** Schematic illustration of the preparation and cancer synergistic phototherapy implementation of GO-808. **C** IR-808 release rate of GO-808 (covalent conjugation) and NGO@808 (non-covalent stacking) in PBS or 10% FBS. **D** Thermal images of PBS (upper row) and GO-808 (bottom row) during 5 min irradiation. **E** In vitro PDT/PTT effects of GO-808 on A549 and Lewis cells at 24 h post laser irradiation. Fluorescence images of Calcein AM-PI co-stained tumor cells. **F** A549 cells treated with IR-808 (10 μM) or NGO-808 (10 μM) in different conditions were imaged by confocal microscope for cell uptake. **G** In vivo tumor-targeted NIR imaging of GO-808 on nude mice bearing A549 tumors and C57 mice bearing Lewis tumors. In vivo images of (a) A549 tumor and (b) Lewis tumor. Ex vivo images of dissected organs and tumors from nude mice (c) and C57 mice (d) at 48 h post injection (from left to right: lung, liver, spleen, heart, kidney, intestine, muscle, tumor). **H** The IR thermal imaging of (a) A549 tumor xenografts and (b) Lewis tumor xenografts were recorded after 5 min 808 nm laser irradiation at 48 h post tail vein injection of PBS, IR-808, GO-PEG-BPEI and GO-808. **I** The tumor volumes in A549 tumor (a) and Lewis tumor (b) treated by different conditions [149]

GO-808 prepared by coupling heptamethine indocyanine dye (IR-808, Fig. 3A) with PEG and branched polyethylenimine (BPEI)-modified GO (Fig. 3B) [149] exhibited a better stability than GO@808 obtained by modifying GO–PEG–BPEI with IR-808 via hydrophobic interactions and π–π stacking, which released less than 10% IR-808 in 24 h (Fig. 3C). In PDT and PTT, GO-808 can not only significantly generate intracellular ROS to kill tumor cells through the apoptosis pathway [150, 151], it can sharply increase temperatures from 24 to 58 °C in 5 min of irradiation (Fig. 3D) and thus showed obvious phototoxicity to A549 and Lewis cancer cells (red and green were dead and live cells, respectively) (Fig. 3E). Furthermore, after 24 h of incubation, GO-808 showed stronger fluorescence signal in A549 cells because the drug carrier made PSs aggregate in cancer cells [152, 153] (Fig. 3F). It is also reported that the cell uptake of 808 is related to energy and organic-anion-transporting polypeptides (OATPs) on the surfaces of many tumor cells [154, 155]. Moreover, this material had high targeting ability and preferentially aggregated in tumor cells. As can be seen from NIR imaging figure, A549 tumors in athymic nude mice (Fig. 3Ga), Lewis tumors in C57 BL/6 mice (Fig. 3Gb) were clearly visualized 48 h after the injection. The IR imaging of the anatomical organs also demonstrated that the fluorescence intensity in tumors was considerably stronger than that in other organs (Fig. 3Gc, d). In contrast to other materials, GO-808 accumulated significantly in the two tumors (Fig. 3H). Importantly, after 5 min of NIR laser irradiation, the temperature at GO-808 increased to the temperature that can eliminate cancer cells. In addition, in contrast to those in the groups treated with other materials and nonirradiation groups, GO-808 has the characteristics of complete elimination and no recurrence (Fig. 3I).

CNTs are excellent carrier materials because of their large specific surfaces and high stability [156, 157]. They also have good mitochondrial targeting ability because of their high hydrophobicity. Thus, the combination of ICG and cationic amphiphilic polymer coated CNTs has also been used in PTT. Under 808 nm NIR irradiation, the prepared materials can accurately and efficiently damage mitochondria by producing ROS and increasing temperatures and thus further induce damaged mitochondria to release ROS to kill cells [158].

In addition to GO and CNTs, covalent organic framework (COFs) and UCNPS have been used in combination with phthalocyanine dyes to construct nanomaterials for PTT–PDT combination therapy. COFs are organic crystalline porous materials, its special structure make them an ideal nano-carrier to load PSs and PTAs [159]. ICG can be adsorbed onto COFs through π–π conjugation, and egg albumin can be coated onto COF@ICG surfaces via electrostatic action. This approach prevents molecular accumulation and improves photostability [160]. Multifunctional UCNPs@PDA-ICG nanocomposites synthesized through hydrophobic electrostatic adsorption and π–π stacking can also enhance the antitumor efficacy of PTT–PDT combination therapy [161].

#### Combination with other materials

In addition to the main phototherapy materials mentioned above, some organic molecules introduced into functional materials can provide benefits to the use of cyanine dyes in PTT–PDT combination therapy [162]. For example, HA-modified Prussian blue can effectively improve the stability of ICG [163]. HA is a biocompatible polysaccharide that can specifically bind to the CD44 receptor that is overexpressed on the surfaces of tumor cells. A novel therapeutic nanomaterial HA–PEG–CyI was constructed by connecting HA to the surface of self-assembly pegylated CyI, and it can activate the immune system and cooperate with phototherapy in tumor treatment [164].

The use of the tumor-homing effect to design molecules with improved targetability is also an effective strategy to design cyanine dye carriers for combination therapy. An interesting example is biomimetic NPs (M@PPI–siRNA), which exhibit strong oral tongue squamous cell carcinoma (OTSCC)-targeting ability due to their tumor-homing effect. These materials were prepared by encapsulating poly β-amino ester/poly lactic-coglycolic acid-blended NPs coloaded with ICG and Nrf2-siRNA in homologous OTSCC-specific cell membrane vesicles. Through the PTT effect and inhibiting the expression of subunits, such as Nrf2, the glutamatecysteine ligase catalytic subunit, and the modifier subunit, with important roles in ROS detoxification, M@PPI–siRNA significantly inhibited tumor growth and angiogenesis [165]. Another study inspired by high-density lipoprotein (HDLs) constructed a bionic nanoplatform capable of penetrating deep tumors. HDLs were bound to tumor-specific iRGD peptides, which can promote the tissue penetration of drugs and have the function of tumor targeting and tumor penetration [166], via a cross-linker to obtain a similar α-helix structure that acted as a tissue scaffold to maintain lipid NPs for ICG binding. The encapsulated ICG could generate heat in the local range of PTT and produce sufficient ROS for PDT [167].

### Porphyrin dyes

#### Combination with metal materials

Metals, especially Au, can be combined with various materials to become the carrier of dyes, including Por dyes [168, 169]. Metallized Pors have the favorable properties of high ^1^O_2_ quantum yield and strong fluorescence imaging ability [170]. Many such materials, such as Au nanoshells and Au nanorods, can enhance the stability of Por [171–174]. For example, Au nanorods were used as carriers to synthesize 4-carboxyphenyl Por-conjugated silica-coated Au nanorods (AuNRs@SiO_2_–TCPP) for PTT and PDT co-therapy under 660 and 808 nm laser irradiation. Compared with the water temperature, which increased from 30.7 to 38.8 °C, the temperatures of AuNRs and AuNRs@SiO_2_–TCPP increased from 32.1 to 56.8 °C and 53.6 °C, respectively, under 808 nm laser irradiation because AuNRs strongly absorbed the light at 808 nm and converted it into heat. When AuNRs@SiO_2_–TCPP was used, the total apoptosis rate rose to 57.2% compared with that of the control cells (0.4%). These results indicated that AuNRs@SiO_2_–TCPP has excellent potential for PDT–PTT. Fluorescence images showed that AuNRs@SiO_2_–TCPP can be absorbed into A549 cells and mainly accumulated inside the cytoplasm. In animal experiments, AuNRs@SiO_2_–TCPP showed the significant activity against A549 tumors in female nude mice through PTT/PDT bifunctionalization and resulted in the lowest relative tumor volume. Moreover, AuNRs@SiO_2_–TCPP had good biological safety in vivo. H&E staining images revealed no noticeable organ damage and inflammatory lesions in the treated nude mice [175].

In another example, a core–shell gold nanoparticle was prepared by using Au nanoparticles and 5,10,15,20-tetrakis(pentafluorophenyl)-21H,23H-porphine (PF6) dye based on porphine [176] and formed a dyad through molecular self-assembly. Au nanoparticles can protect PF6 and improve the stability of the dye. PF6 dyes in shell core assured the effectiveness of singlet oxygen production. The experimental results demonstrated that this design improved the PDT and PTT therapy [177].

Porphyrin dye is also often combined with zinc to form PDT/PTT materials [178–180]. Because the lysosome in tumor cells has an acidic environment (pH 4.5–5.0), glycolysis can trigger the acidic tumor microenvironment under hypoxia to construct a specific pH responsive target PTT [181–184]. A pH-responsive zinc (II) metallized porphyrin was designed to amplify cancer PDT/PTT. Under single 660 nm laser irradiation, the tumor disappeared completely without side effects [170].

In addition to Zn and Au, other metallic materials, such as Fe, Cu, and Pt, are used in combined phototherapy [185–189]. For example, CS-coated multifunctional metal–organic NPs (Fe–TCPP@CS NPs) were designed to combine electrostatic targeting with PDT and PTT antimicrobial therapies. Tetrakis(4-carboxyphenyl) Por (TCPP) was used as a PS to generate ^1^O_2_ under irradiation. Fe_3_O clusters can realize low-temperature PTT therapy and avoid PS self-aggregation. CS was used as the outer layer of Fe–TCPP to enhance dispersity and improve electrostatic binding with the bacterial cell membrane. Under light irradiation, Fe–TCPP@CS NPs could produce sufficient ROS and heat to kill a variety of harmful bacteria [190]. Another example is PCN 224, which was fabricated through the connection between Zr6 clusters and TCPP [191–193] to enable combined PDT and PTT. Cu^2+^ was introduced into the Por ring of PCN 224. This approach strengthened the photothermal effect of metal–organic frameworks (MOFs) due to the light absorption derived from the d–d transition [194, 195]. Under 660 nm light irradiation, the Cu^2+^ can capture electrons, thus suppressing electron–hole recombination and accelerating carriers transfer to enhance ROS yields. MOFs doped with 10% Cu^2+^ had the best antibacterial effect of 99.71% against *Staphylococcus aureus* within 20 min due to the synergistic effects of heat and ROS [196].

#### Combination with non-metallic materials

Similar to cyanine dyes, Por dyes have been used with metallic and nonmetallic materials in PTT–PDT combination therapy [197]. Graphene, COFs, and silica are commonly used materials [198, 199]. GO is a highly popular material and has myriad applications in combination therapy [200]. A unique one-dimensional linear graphene nanoribbon (GNR) superstructure coated with cationic Por (Pp4N) NPs was constructed through the supramolecular self-assembly of GNRs and Pp4N. The designed materials had excellent ROS yield in PDT and showed high temperature in PTT under 660 and 808 nm irradiation [201]. A novel GO–C_60_ hybrid with a good solubility in different environments, including physiological solutions, was constructed through the progressive conjugation of hydrophilic methoxy PEG and monosubstituted fullerene C_60_. The binding of GO to C_60_ did not reduce the PTT properties of GO but instead activated the ability of C_60_ to generate ^1^O_2_ in the aqueous solution in the NIR region. In addition, the GO–C_60_ hybrid had good ROS generation ability. It showed unlimited potential in inhibiting cancer cells through synergistic effects [202].

Although PSs and PTAs have excellent PDT and PTT properties, their biological applications are severely limited by their poor water solubility. To overcome this problem, COFs were introduced and used as an ideal nanocarrier for the delivery of hydrophobic organic molecules, PSs, and PTAs due to their free active end groups and large inner pores [203]. Based on this concept, VONc@COF–Por, a COF nanoplatform for PDT and PTT with surface-decorated Por and encapsulated naphthalocyanine (VONc), was successfully prepared by covalently grafting Por PS and noncovalently encapsulating naphthalocyanine. VONc@COF–Por not only had high ^1^O_2_ production and strong photothermal conversion ability (55.9% of conversion efficiency) but also was internalized and localized in the cytoplasm by MCF-7 cells through endocytosis. Moreover, VONc@COF–Por showed low toxicity and good biocompatibility. The viability of MCF-7 cells under PDT–PTT co-therapy with VONc@COF–Por sharply declined to 16.5% under 808 nm laser irradiation. The IC_50_ was only 42 μg/mL, which was considerably better than the IC_50_ of PDT or PTT monotherapy. Furthermore, calcein-AM/PI double staining showed that co-therapy had outstanding effects. The percent of dead cells enhanced from 41% under PDT monotherapy to 75% under PTT monotherapy and finally reached 94% under PDT–PTT co-therapy [204]. These results demonstrated that OSMDs can be combined with COF to construct an effective PDT–PTT treatment platform.

#### Combination with other materials

The combination of Por dyes and other materials also plays an important role in PDT–PTT therapy [205–208]. For example, a new Por compound (TPAPor) was synthesized through the covalent conjugation of Por and triphenylamine. The conjugated structure caused redshift in the NIR region. Subsequently, amphiphilic organic molecules were self-assembled into NPs (TPAP or NPs) with uniform particle sizes (approximately 80 nm) that enhanced NIR absorption and had good biocompatibility [209]. Core–shell nanohybrids (PB@PCN) were prepared by coating Prussian blue (PB) NPs with zirconium–Por (PCN) shells. The influence of PCN shell on the photothermal properties of the core is very small. Therefore, PB@PCN showed good photothermal conversion ability, which can increase the temperature from 32 to 55 °C within 10 min. This rate increased with the increase in concentration. Moreover, PB@PCN possessed a good ROS yield [210]. The combination of these materials and Por can strengthen biocompatibility, NIR absorption, and PTT effect; produce high yields of ^1^O_2_; and enhance water solubility. These effects confer numerous benefits to PDT–PTT combination treatment.

### Rhodamine dyes

**Fig. 4:**
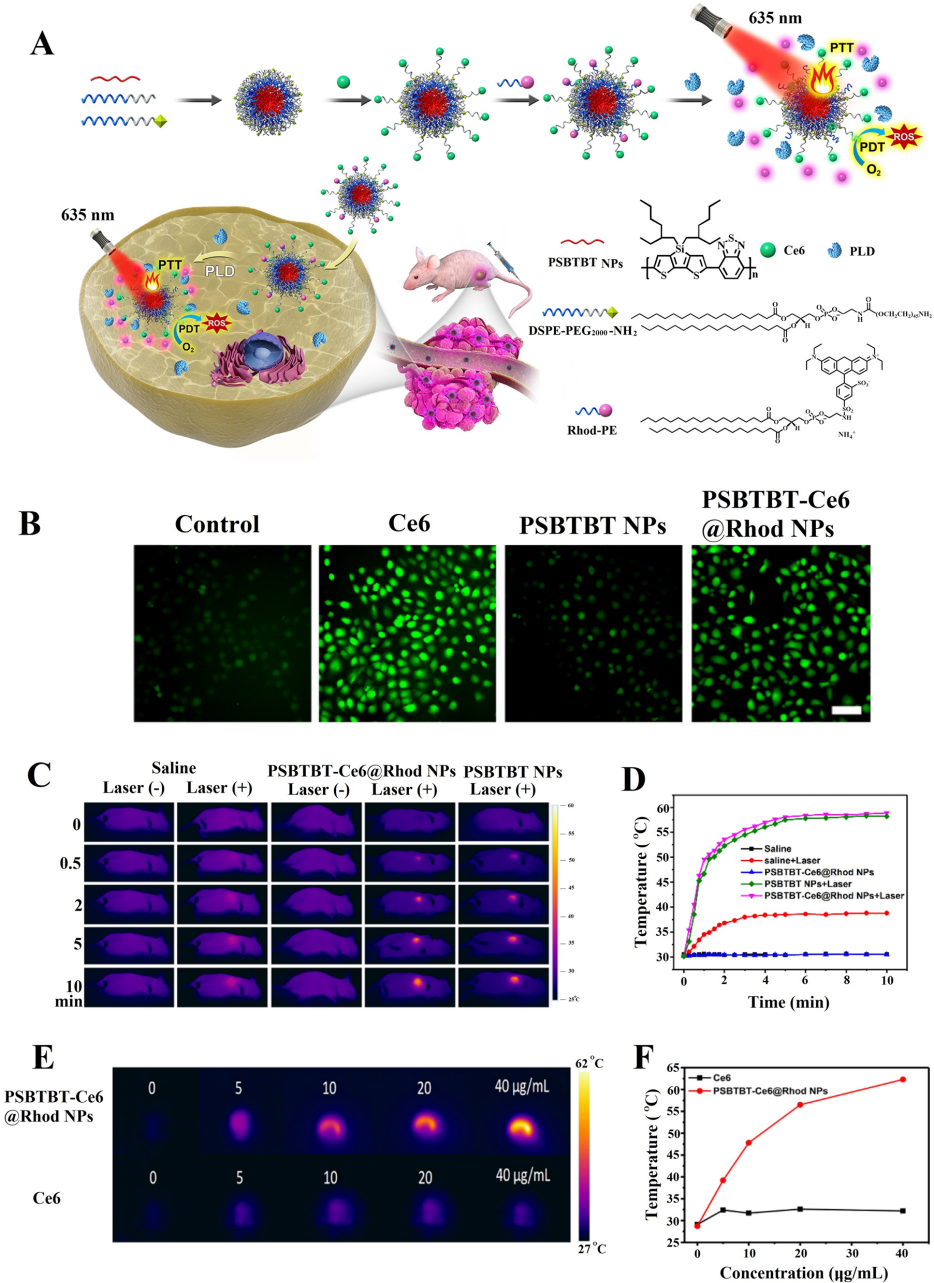
PSBTBT-Ce6@Rhod NPs nanoplatform for precise PTT/PDT co-therapy. **A** Schematic illustration of PLD-activatable tumor image and PTT/PDT co-therapy. **B** Singlet oxygen level indicated by DCF-DA staining in MCF-7 cells that incubated with free Ce6, PSBTBT NPs, and PSBTBT-Ce6@Rhod NPs. Green color represents fluorescence of DCF-DA. **C** IR thermal images of tumor-bearing mice in different groups after NIR laser irradiation for 10 min. **D** Tumor temperature changes treated with saline, PSBTBT-Ce6@Rhod NPs and PSBTBT NPs under laser irradiation. **E** Thermal images and **F** quantitative temperature change curve of PSBTBT-Ce6@Rhod NPs in PBS buffer solution [215]

Although rhodamine is widely used in fluorescence imaging [211, 212] and phototherapy [213, 214], usually image-guided PDT or PTT therapy, its technology for PDT–PTT co-therapy remains insufficiently mature with only a few specific research examples. A multifunctional phospholipid modified semiconductor polymer nanoplatform (PSBTBT–Ce6@Rhod NPs) was developed by using phospholipase, Ce6, and rhodamine B. PSBTBT-Ce6@Rhod NPs can be used for proportional phospholipase D detection and combined PTT–PDT processing (Fig. 4A) [215]. They can produce ^1^O_2_ to kill MCF-7 cells and showed strong fluorescence, which was monitored by employing 2ʹ,7ʹ-dichlorodihydrofluorescein diacetate (DCF-DA) as the fluorescence indicator (Fig. 4B) [216, 217]. Moreover, PSBTBT-Ce6@Rhod NPs not only rapidly heated up to 58 °C in 5 min under laser irradiation, thus showing good photothermal conversion efficiency (Fig. 4C, D), they also had good photothermal stability. Their photothermal effect was still the same even after five times of heating/cooling. In addition, the photothermal images showed the signal continued to increase as the concentration increased (Fig. 4E, F).

### Squarine dyes

SQ dye, one of the most promising NIR fluorescent dyes, has broad application prospects in PTT–PDT co-therapy due to its high absorption coefficient, bright fluorescence, and photostability. It can be combined with Cy7, BSA, and geldanamycin (GM) to prepare BSA/Cy7–SQ/GM NPs for the acceleration of cancer cell apoptosis via NIR laser irradiation. Given that low-intensity NIR (650–900 nm) radiation had deep penetration and minimal invasion to tissues [218], Cy7–SQ was designed as a multifunctional NIR agent through a covalent disulfide linkage between a PTA (Cy7) and a PS (SQ) to improve their photostability and thermal stability. The materials could generate ROS and heat for efficient synergistic PDT and PTT [219].

### Boron dipyrrin dyes

Functional group modification is a good method to improve the PTT efficiency and ROS yield of BODIPY dyes [220–222]. For example, PTT conversion efficiency was improved by modifying a BODIPY dye with trimethylamine into BDPTPA [223]. BDPTPA not only had good cell uptake behavior and can be used for cell imaging in vitro. It also possessed high ^1^O_2_ production ability (35.2%). In addition, strong green fluorescence was observed in the investigation of ROS generation by using DCF-DA, indicating that BDPTPA can produce strong ^1^O_2_. Moreover, after BDPTPA injection, the fluorescence signal of the tumor increased rapidly, and fluorescence intensity reached the highest level after 6 h. BDPTPA was mainly distributed in the tumors, livers, and kidneys of the killed mice. Furthermore, BDPTPA had high photothermal conversion efficiency (52.6%) and rapidly increased the tumor surface temperature to 57 °C within 8 min under irradiation. These results indicated that BDPTPA is a potential candidate for synergistic PDT–PTT therapy.

BODIPY dyes can also be coated with nonmetallic materials. Aza-BODIPY-based PS and MeOABBr were encapsulated with PEG-FA and PEG-triphenylphosphonate to obtain tumor and mitochondrial dual-targeting NPs (FMAB NPs). FMAB NPs can generate ROS under 730 nm laser irradiation. Furthermore, after treatment with FMAB NPs, the tumor tissue temperature increased from 35.0 to 48.1 °C within 8 min after irradiation. The results of photoacoustic (PA) imaging showed that the signal intensity of PA increased significantly at 24 h after intravenous injection, indicating that FMAB NPs had high tumor specificity [224]. In addition, the real-time monitoring of the accumulation of FMAB NPs in vivo showed that the fluorescence of FMAB NPs reached the maximum at 4–8 h after injection. The collected tumors and other organs revealed that fluorescence intensity was the highest at the tumor site, and only weak fluorescence was found in the lung, heart, liver, kidney and spleen. The results proved once again that FMAB NPs had excellent tumor targeting ability [225]. In addition, metal has been used to bind BODIPY dyes. Aza-BODIPY probe-modified mesoporous TiO_2_ NPs strengthened PDT and PTT under single-wavelength NIR laser irradiation [226].

## Image-guided phototherapy of NIR-OSMDs

Phototherapy is an alternative to surgical resection for cancer treatment. However, it still has its limitations. For example, when removing cancerous tissue, there may inevitably be side effects, including systemic toxicity or harm to healthy tissues. With the progress of science and technology, other therapeutic methods can now be combined with phototherapy. The integration of diagnosis and therapeutics can realize visual diagnosis and accurate treatment [227]. Image-guided PDT–PTT synergetic therapy is not only of great significance in improving the accuracy and efficacy of cancer treatment, but also in minimizing damage to normal tissues as much as possible. Imaging-guided phototherapy has been realized owing to the various imaging capabilities of NIR-OSMDs (Table 1). PA imaging, FL imaging, and magnetic resonance (MR) imaging, the main imaging technologies that are combined with phototherapy, have made an important contribution to the increased accuracy of phototherapy.

### Photoacoustic imaging

PA imaging is a noninvasive cancer imaging technique with the advantage of high contrast, sensitivity, and spatial resolution [228, 229]. A probe that can produce a strong signal has to be developed to real-time illustrate the relationship between probe states and pathological processes to obtain PA imaging [230, 231]. The combination of PDT and PTT usually uses composites, the addition of various components enables some photoactive agents to perform PA imaging and strengthens PDT–PTT treatment. Examples of PDT–PTT therapeutic systems involving OSMDs exist [232, 233]. For example, Por-based COF NPs (COF-366 NPs) were synthesized to control the orderly spatial arrangement of photoactive building units for providing PA imaging-guided PDT–PTT therapy. COF-366 NPs can not only reduce Por quenching due to the increased distance between molecules [234], they also exhibit biosafety due to their metal-free synthesis process and degradability due to their dynamic reversible bond. Furthermore, given the broadened absorption spectrum of the conjugated structure, COF-366 can be used as the PS and PTA to generate heat and ROS under single-wavelength light irradiation. When DCF-DA was used as an indicator, 4T1 cells treated with COF-366 presented strong and obvious green fluorescence signals under 635 nm laser irradiation, indicating ROS production in the cells. Moreover, COF-366 had strong thermal conversion ability and can rapidly increase the temperature from 36.2 to 60.4 °C within 5 min. Furthermore, the enhanced absorption in the NIR region made COF-366 NPs capable of PA imaging, which can provide distribution images in biological tissues [235–237]. In tumors, it reached its maximum value at 12 h after the intravenous injection. The above properties enable COF-366 NPs to realize precise PDT-PTT treatment in vivo [238].

### Fluorescence imaging

FL imaging has become a basic tool for biomedical applications and is expected to visualize biological structures through connective tissues with thicknesses of 5–10 mm [239, 240]. Cyanine dyes, especially ICG and its derivatives, have wide applications in FL imaging [240–243]. For example, the ICG derivative Cy7 was modified with heavy atomic iodine to form the new low-toxicity NIR dye CyI. CyI enhanced ROS and heat generation to induce the apoptosis and inhibition of deep HepG2 tumor cells while maintaining FL imaging properties for noninvasive in vivo imaging. Therefore, FL imaging-guided PDT–PTT based on CyI is a new method to treat cancer cells in deep tissues [244]. However, NIR-triggered cyanine dyes for tumor treatment usually have low FL intensity and ^1^O_2_ production efficiency. Plasmonic enhancement is one of the most effective methods for overcoming these shortcomings [245]. The unique electromagnetic field on the surfaces of noble metals can considerably affect the excitation rate, emissivity, or nonemissivity of nearby PSs, resulting in changes in FL and ^1^O_2_ [246]. AuNRs@SiO_2_–IR795 nanocomposites based on metal-enhanced fluorescence (MEF) effects were designed and prepared by using AuNRs@SiO_2_ and NIR-fluorescent IR795 (derived from IR783 dye) for FL imaging guided-PDT–PTT co-therapy. The enhanced FL intensity of AuNRs@SiO_2_–IR795 can achieve highly efficient bioimaging. Compared with IR795 alone, AuNRs@SiO_2_–IR795 emitted a clear FL signal in the cytoplasm with a low dosage of IR795 dye, and the FL intensity was significantly enhanced by up to 51.7. Moreover, under 808 nm laser treatment for 30 min, the ^1^O_2_ efficiency of AuNRs@SiO_2_–IR795 was up to 6.3-fold higher than that of an equal amount of IR795 (0.4 μM). In addition, this material had excellent photothermal conversion efficiency and can quickly increase the temperature from 29.2 to 86.5 °C within 5 min. These results demonstrated that this material has high potential for application in FL imaging-guided PDT–PTT treatment platforms [247].

### Magnetic resonance imaging

MR imaging is an imaging method that aims to show the regional and time-varying changes in brain metabolism [248]. With the development of research, it has become gradually used in imaging the tumors of the central nervous system, limbs, and abdomen with excellent structural details [249, 250]. MR imaging can provide three-dimensional topographical data with high spatial resolution, realize organ structure contrast, and output the information of disease tissue in real time [251]. It can also be used in conjunction with PDT–PTT therapy for visual guidance [185, 252]. MR imaging-guided applications in PDT–PTT therapy based on OSMDs are also common [253–256].

For example, MR imaging was used to guide PDT–PTT co-therapy for cancer treatment with increased accuracy and effectiveness. A yolk–shell nanostructure of FeS_2_@C–PEG (200 nm) was prepared. It showed strong NIR absorption and high PTT conversion (42.3%). Cells cultured with FeS_2_@C–PEG exhibited green fluorescence that increased with culture time, indicating that a large number of ROS were generated. In the hypoxic state, FeS_2_@C–PEG can also induce ROS production. Moreover, the MR property of FeS_2_@C–PEG exhibited the linear relationship between Fe ion concentration and transverse relaxation was good. The tumor location on the image considerably darkened after the injection of this material, demonstrating the contrast enhancement of the tumor region in MR imaging. During the treatment of 4T1 tumor-bearing mice with FeS_2_@C–PEG, the temperature of the tumor site increased to 46.8 °C after 6 min of NIR irradiation. After 15 days of treatment, compared with various controls, the FeS_2_@C–ICG–PEG group exhibited the smallest tumor size. This result indicated that the synergistic effect of PTT and PDT enhanced the anticancer effect [253]. In another study, GNRs@BPP–Gd with bare toxicity based on Au nanorods and Gd-chelated Por–BSA complexes was synthesized through a simple and environmentally friendly method to guide PDT–PTT therapy through MR imaging. Paramagnetic Gd complexes were introduced into PS and Por–protein complexes as a T1-weighted MR imaging probe to guide treatment because they are widely applied in the clinic as T1 contrast agents that increase the sensitivity of MR imaging [251]. Subsequent cell uptake experiments confirmed that GNRs@BPP–Gd was effectively internalized into cancer cells for the complete ablation of tumors, revealing a considerable antitumor effect [256].

### Multimodal imaging

Finding a single imaging method that meets the requirements of modern therapy has become difficult with the maturation and application of various imaging technologies [257]. Therefore, multimodal imaging technology is integrated and designed not only to improve the localization and targeting of diseased tissues but also to monitor and control treatments [258–261]. A multimodal imaging theranostic system for imaging-guided PDT–PTT based on OSMDs has also been gradually established and continuously developed [262, 263]. PA imaging and FL imaging are commonly combined. For example, one study constructed a single-light triggered ICG-loaded PEGylation silver NP core/polyaniline shell (Ag@PANI) nanocomposite (ICG–Ag@PANI) for PA/FL imaging-guided enhanced synergistic PDT–PTT treatment. PA/FL dual-modal imaging was used to track the accumulation and distribution of ICG–Ag@PANI in tumors based on the enhanced EPR effect, which provided precise guidance to treatment [264]. Another work focused on the programmed assembly of human serum albumin (HSA)–ICG NPs through intermolecular disulfide conjugations. In this research, dual-modal PA/FL imaging and spectrum-resolved technology based on ICG were applied to identify the tumor precisely. Meanwhile, the HSA–ICG NPs effectively induced ROS and produced high temperature simultaneously for synergistic PDT–PTT treatment [263].

**Fig. 5:**
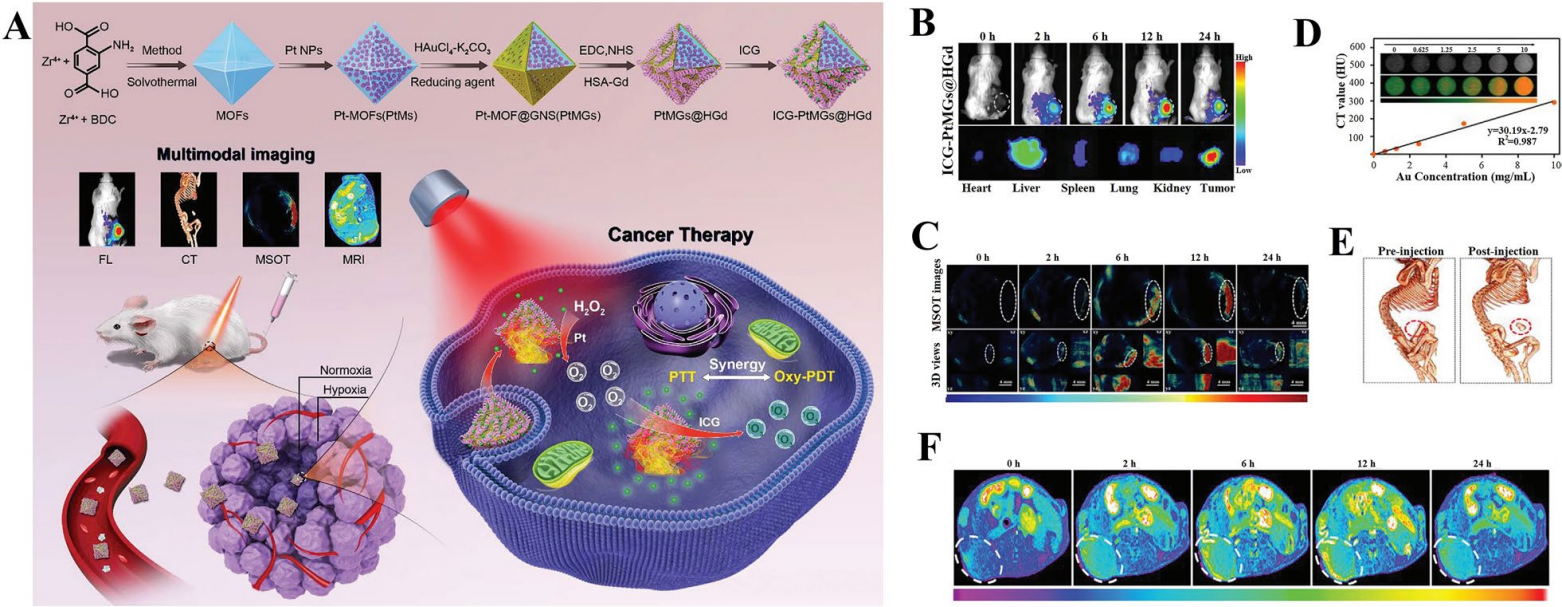
ICG-PtMGs@HGd nanoplatforms for multimodal imaging-guided synergistic phototherapy. **A** Scheme of the ICG-PtMGs@HGd nanoplatforms as H_2_O_2_-driven oxygenator for FL/MOST/CT/MR multimodal imaging guided enhanced PDT and PTT synergistic therapy in a solid tumor. **B** Real-time fluorescent images of 4T1 tumor-bearing mice at different time points after administration of ICG-PtMGs@HGd; the bottom panel shows the ex vivo images examined at 24 h postinjection. **C** The MSOT and 3D orthogonal MSOT images of 4T1 tumor-bearing mice after being intravenously injected with ICG-PtMGs@HGd nanoparticles at different time points. **D** CT signal intensity linearly fits to the concentration of ICG-PtMGs@HGd aqueous solutions; inset: the corresponding CT images. **E** 3D reconstructed CT images before and 12 h after injection of ICG-PtMGs@HGd. **F** T1-weighted MR images after intravenous injection with ICG-PtMGs@HGd at predesigned time points [262]

Furthermore, the involvement of additional imaging techniques can enable the investigation of lesions based on different mechanisms. Such investigations can help guide treatment accurately. Octahedral Au nanoshells were grafted onto the template of platinum nanozyme-decorated MOFs, then further functionalized with HSA-chelated Gd (HGd) and loaded with ICG to construct nanocomposites (ICG–PtMGs@HGd) (Fig. 5A) [262]. Similar to other nanotherapeutic platforms constructed with OSMDs, this material had good ROS generation and thermal conversion abilities to achieve a synergistic PDT–PTT effect. Importantly, it can also realize multimodal imaging to provide complementary anatomical and functional information that is crucial for accurate tumor detection and treatment. ICG is an ideal reagent for FL imaging because of its fluorescence. This ability was strengthened after the formation of the nanoplatform. At 12 h after injection, the nanoplatform accumulated at large quantities and remained for a long time in the tumor. The intensity of fluorescence signals in the tumor was considerably higher than that in metabolic organs, such as the liver, spleen, and kidney (Fig. 5B). Given its strong NIR absorption and photothermal conversion ability, ICG–PtMGs@HGd also had excellent contrast multispectral optoacoustic tomography (MSOT) imaging ability. ICG in the material induced MSOT signals that peaked at 12 h after injection (Fig. 5C). Moreover, the nanoplatform can be used for CT imaging because of the high X-ray attenuation by the Au atom [265]. The brightness of CT imaging had a linear relationship with the concentration of Au (Fig. 5D). Similarly, good CT imaging was obtained at 12 h after injection (Fig. 5E). In addition, the ability of the MR signal of the nanoplatform to provide clear information on soft structures without ionizing radiation was superior to that of Gd pentanoic acid, a clinical MR contrast agent [266–268]. The clear outline of the tumor was depicted in the brightest image with sharp edges of live T1-weight MR imaging at 12 h after intravenous injection (Fig. 5F). These pieces of evidence suggested that ICG–PtMGs@HGd can be used as a versatile reagent for multimodal imaging to provide additional information on tumors over a wide spectrum.

A nanoplatform based on CuS and Por-modified CS-coated Fe_3_O_4_ NPs (FCCP NPs) with a highly reactive peroxidase (Fe_3_O_4_ structure) [269, 270] was designed to convert endogenous H_2_O_2_ into •OH as a therapeutic element to overcome the hypoxia problem encountered in PDT. This nanoplatform not only solved the problem of hypoxia in the treatment process but also enable combined PDT–PTT under the guidance of multimodal imaging [271]. The realization of multimodal imaging mainly depended on the tumor-targeting ability of FCCP after intravenous injection. In mice, the temperature of the tumor surface rose rapidly to 50 °C under 980 nm laser irradiation after the injection of FCCP. Such good photothermal conversion performance enabled the use of the nanoplatform for photothermal imaging in vivo, thus providing a potential tool for distinguishing tumors from normal tissues [272]. Furthermore, when the maximum excitation wavelength was 410 nm, the synthesized Por showed strong photoluminescence (PL) at 671 nm, which was conducive to PL imaging [273]. Ex vivo PL imaging at 12 h after injection showed that FCCP accumulated mainly in tumors, as well as in the liver, As a result of good targeting. In MR imaging, the FCCP NPs enhanced magnetic resonance contrast. In addition, the tumor tissues of mice injected with FCCP exhibited strong PA signals. These results demonstrated the multimodal imaging ability of FCCP NPs, which is a powerful tool for diagnosing tumor tissues [271].

## Synergistic chemotherapy and phototherapy with OSMDs

Combining anticancer drugs with targeted therapy is another highly promising choice for further improving the efficacy of PDT–PTT synergistic therapy [274]. Some studies had introduced imaging guidance, which increased the effectiveness of treatment (Table 1) [275–277].

### Synergistic chemo/PDT/PTT

Anticancer drugs, OSMDs, and a variety of advanced functional materials are combined to form therapeutic nanoplatforms that generally have targetability. When the nanoplatforms reached the target, anticancer drugs and dyes can be released under internal stimulation (e.g., pH variations [278], catalytic action of enzyme [279], and redox gradients [280]), and external stimulation (e.g., light [281], temperature [282], ultrasonic waves [283], and magnetic field [284]). The released drugs can kill cancer cells, and the dyes and nanomaterials are conducive to the local generation of ROS and heat to achieve the PDT–PTT effect. The commonly used drugs include doxorubicin (DOX) [285, 286], cisplatin [287, 288], paclitaxel [289], camptothecin [290], wedelolactone [291], sorafenib [292], piperlongumine [293], and methotrexate [294], and ICG is the most widely studied dye.

DOX is the most widely used anticancer drug in chemo–PDT–PTT. Many specific examples for the use of this drug are available [295–304]. Au nanocages (AuNCs) with porous walls and hollow interiors are novel platforms for cancer therapy. They can not only be used as drug reservoirs [305], they also have the ability for photothermic conversion [306]. After absorbing the energy of irradiated light, AuNCs can convert the energy of excited photons into heat energy and release drugs simultaneously to realize PTT–chemo co-therapy [307]. In one study, the NIR-triggered chemotherapeutic agent DOX, a PS, and ICG were filled into the interior of AuNCs to fabricate the nanoplatform DOX/ICG@biotin–PEG–AuNC–PCM, which can be used for chemo/PDT–PTT co-therapy. AuNCs can be taken up by MCF-7/ADR cells via endocytosis and quickly co-release DOX and ICG under 808 nm NIR irradiation. Chemo–PDT–PTT co-therapy was realized under the synergistic effects of DOX on cytotoxicity, ICG on ROS, and AuNCs on heat [301]. An apoptosis experiment also verified the good therapeutic effect of combination chemotherapy, and the total percentage of apoptosis in the DOX/ICG@biotin–PEG–AuNC–PCM group was 97.17%, which was drastically higher than that in the other control groups. Meanwhile, PDT and PTT reversed DOX resistance [308].

In addition, 5,10,15,20-Tetrakis(1-methylpyridinium-4-yl) Por (TMPyP4) has been applied in addition to the classical OSMD ICG. In one work, DOX and TMPyP4 were inserted into a DNA assembly immobilized on AuNRs. These drugs were delivered to the target cells effectively, and then released under light irradiation, thus forming a synergistic effect of chemotherapy and phototherapy for cancer treatment [300].

### Image-assisted tri-modal therapy

**Fig. 6:**
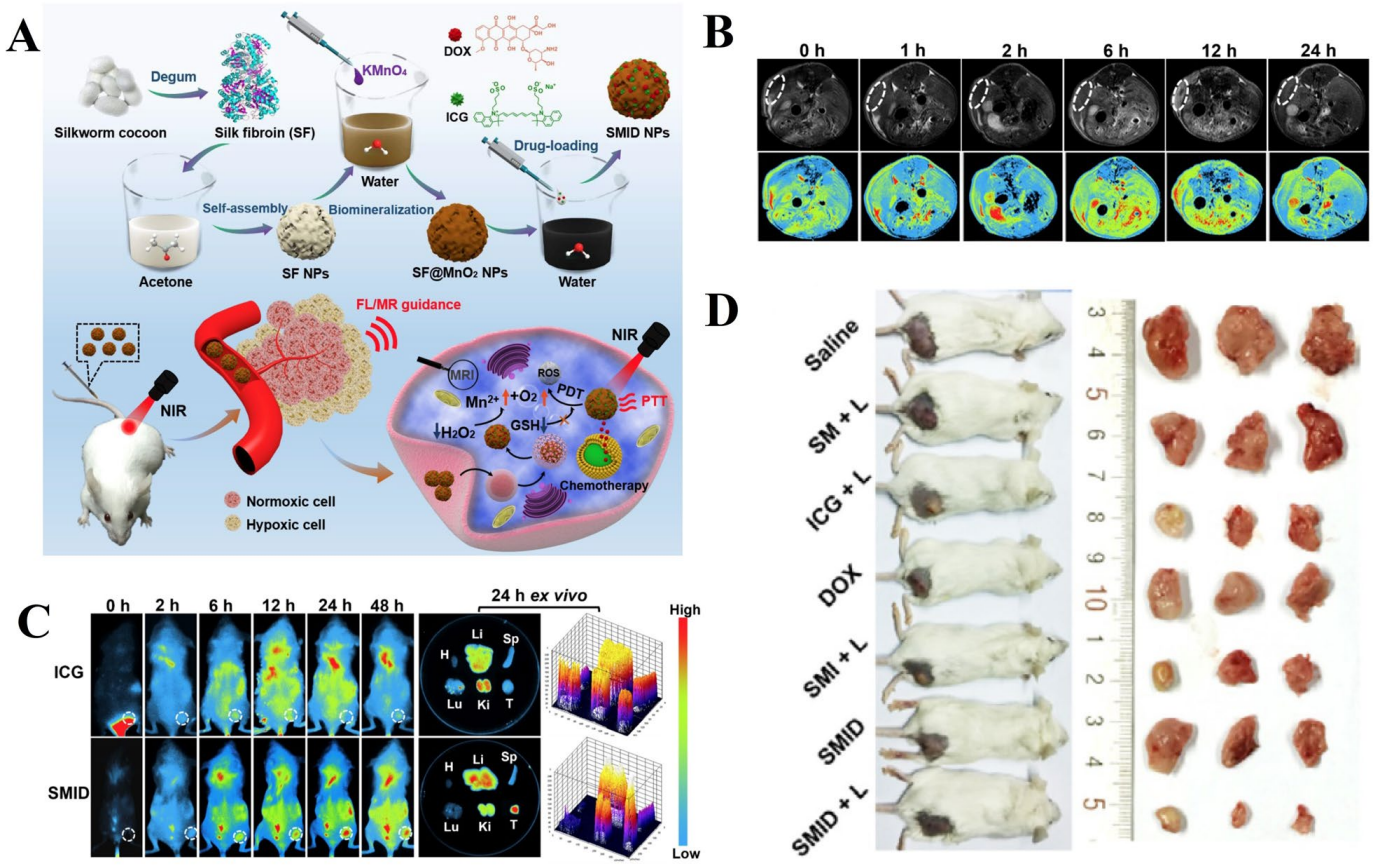
Biomineralization-inspired crystallization of nanganese oxide on silk fibroin nanoparticles for in vivo MR/FL imaging-assisted tri-modal therapy of Cancer. **A** Schematic of SF@MnO2/ICG/DOX (SMID) nanoplatform for in vivo MR/FL imaging-assisted tri-modal therapy of cancer. **B** T1-weighted greyscale and pseudo-color MR images of 4T1 tumor-bearing mice (transverse plane sections) before and after intravenous injection of SMID nanoparticles (equivalent ICG concentration: 3 mg kg^−1^). White dashed circles denote tumor locations. **C** Biodistribution of SMID nanoparticles in vivo: fluorescence images of 4T1 tumor-bearing mice taken at different time points after intravenous injection of ICG or SMID nanoparticles (equivalent ICG concentration: 3 mg kg^−1^), and ex vivo fluorescence images and corresponding optical intensity of tumor and major organs (T, Li, Sp, Ki, H, and Lu denote tumor, liver, spleen, kidney, heart, and lung, respectively) dissected at 24 h post-injection. **D** Photographs of treated mice and the corresponding tumors excised at day 14 post-injection [276]

Imaging technologies are also an important guide in chemo–PDT–PTT co-therapy. In a captivating study, regenerated silk fibroin (SF)@MnO_2_ was prepared by using a one-pot fabrication technique. Then, ICG and DOX were co-immobilized into SF@MnO_2_ NPs to form SF@MnO_2_/ICG/DOX (SMID) (Fig. 6A) [298]. Each component of this NP had a unique role. MnO_2_ can react with H_2_O_2_ to generate O_2_ for PDT. In addition, the Mn atom in MnO_2_ can improve the contrast of MR imaging by strengthening the relaxation of water protons. The MR imaging of 4T1 tumor-bearing mice injected with SMID NPs showed that the imaging contrast at the tumor site enhanced over 12 h (Fig. 6B). ICG can not only promote the production of ROS, it can also enable the in vivo FL imaging of tumors [298, 309]. In the SMID treatment group, the FL intensity of the tumor area gradually increased (6–24 h) and peaked at 24 h, showing that NPs were enriched in the tumor by EPR effect. In vivo FL/MR imaging demonstrated that the NPs were effectively aggregated in the tumor through the EPR effect (Fig. 6C). PH-dependent DOX release was favored in the acidic tumor microenvironment. ICG and SF@MnO_2_ played important roles in PTT. Therefore, the multifunctional SF@MnO_2_/ICG/DOX realized MR/FL dual-modal imaging-guided PDT–PTT–chemotherapy. Tumor-bearing mice were divided into six groups randomly and received different therapy. The results of tumor anatomical size and weight at day 14 showed that the SMID-mediated trimodal co-therapy got the best tumor inhibition. The tumor inhibition rate under this treatment was 89.6%, which was considerably better than that under other treatments (Fig. 6D).

In another work, the multifunctional drug delivery system DOX-loaded folate receptor α (MTX)-decorated self-assembled Zn phthalocyanine–soybean phospholipid (ZnPc–SPC) (DZSM) was prepared for the precise PA/FL imaging-guided PDT–PTT treatment of tumors. In this system, the ZnPc–SPC complex showed PTT, PDT, PA and low-background FL properties. PA intensity gradually increased after injection. At approximately 2 h, DZSM accumulation at the tumor site in 4T1 tumor-bearing BALB/c nude mice reached the maximum and the signal intensity gradually weakened likely due to the cell uptake and decomposition of DZSM. Given that ZnPc had self-fluorescence characteristics, DZSM can be used as a tracker for FL imaging. As a result of the targeting ability of MTX, DZSM tended to accumulate in tumor tissue instead of in the control tissue, and the FL signal gradually appeared in the tumor area at 12 h after injection. The experimental results indicated that DZSM not only exhibited high selectivity for the overexpression of MTX in tumor cells but also had excellent switchable PA/FL imaging ability. DZSM showed significant potential for chemo–PTT–PDT co-therapy given its obvious inhibitory effect on tumor growth in mice and superiority over other controls under 638 nm laser irradiation [275].

## Conclusion and perspective

The successful application of nanotechnology in phototherapy avoids the adverse effects of surgery and chemotherapy on the patient's body. In the continuous improvement process of nanomaterials, their toxic side effects have been reduced and their biocompatibility has been improved. While achieving phototherapy, loading drugs into nanomaterials and implementing image-guided operations have further improved the accuracy and effectiveness of tumor treatment, providing assurance for cancer treatment.

All in all, photoinduction therapy has an increasingly prominent advantage in the war against cancer due to its noninvasiveness and low side effects. NIR-OSMDs have good photothermal effect and ROS production in the range of NIR light. On this basis, NIR-OSMDs have attracted increasing attention for their low toxicity and good biocompatibility. Notably, synergistic PDT–PTT treatment with OSMDs can produce a superposition effect that is greater than the expected sum of each treatment, leading to the overall improvement in therapeutic effect and the best therapeutic effect of completely eradicating malignant solid tumors. NIR-OSMDs can also be combined with chemotherapy, imaging, and other technologies in addition to PDT–PTT in the treatment of tumors as summarized in this review. However, the clinical research, evaluation, and application of PDT–PTT combination therapy based on NIR-OSMDs remains in their infancy. The following problems need to be solved in the future to optimize penetration and greatly accelerate translational bench-to-bedside application.

As a result of reduced light scattering and absorption in the NIR-II region, phototherapy in the NIR-II biowindow is superior to that in the NIR-I biowindow in terms of maximum permissible exposure and deep penetration [11]. PTT or PDT in the NIR-II range can reach and resect deeply buried tumors. However, the current NIR absorption range of OSMDs is mostly in the NIR-I range, and only several small molecular dyes with added functional groups have been studied. Therefore, new synthesis routes and methods should be developed to extend the absorption range of OSMDs to the NIR-II absorption range to improve therapeutic effects. Meanwhile, the structure–activity relationship of dye molecules should be further investigated to optimize the photostability and quantum efficiency of dye molecules, and the interaction of dyes with biomolecules, organelles and cells should be further studied to optimize the molecular structure, enhance the specificity, and reduce the biological toxicity of dyes.

OSMD-based treatment also has numerous problems. The first is the generation of ROS. Generating sufficient ROS is a key issue in PDT. At present, a large number of OSMD nanomaterials have been used to treat advanced tumor hypoxia and improve the efficacy of PDT. However, the relationship between the retention time and spatial distribution of O and the enhancement of the antitumor effect remains to be further studied. Meanwhile, the influence of dye dosage and NIR radiation intensity on the temperature produced by the photothermal effect in PTT is rarely studied [310]. Temperature is also a key problem. Excessively low temperatures cannot kill cancer cells, whereas excessively high temperatures can burn skin and damage healthy cells. Therefore, solving this problem is urgently needed. Cooperative multimodal treatment based on OSMDs in combination with PDT, PTT, and chemotherapy is better than a single treatment. The premise of chemo–PDT–PTT combination therapy is to form a drug-release platform that is composed of drug molecules and carriers. Under the action of the immune and excretory systems, such a platform inevitably has a larger volume and shorter clearance time than the drug molecules themselves. Additional injections are needed in such cases to ensure that sufficient drugs are present at the tumor site to make the treatment effective. Therefore, controlling the size and surface properties of OSMD drug carriers is necessary to prolong the clearance time of drug carriers, enhance the targeting of tumor sites, further improve the stability and half-life of dyes in blood circulation, build a long-cycle drug delivery system, and improve bioavailability [139]. At the same time, the easy interaction of OSMDs with biological molecules, such as proteins, in the body during biological imaging can affect their optical properties and physiological functions and subsequently influence the imaging effect. However, the research in this field is still lacking and needs to be strengthened.

Achieving an excellent synergistic effect through the reasonable selection and design of OSMD materials for collaborative photoinduced therapy is an urgent problem to be solved at present. Thus far, only the role of heat in facilitating drug release from carriers has been demonstrated. The synergistic effect of PDT/chemotherapy and PTT/PDT remains unclear and need to be developed by building nanotherapy platforms. In addition, the influence of particle size, surface properties, water solubility, and other factors on the clearance time, targeting, and biological distribution of OSMD nanoplatforms have a great influence on clearance. Finally, the operational parameters of in vitro and in vivo studies need to be standardized. These parameters include light and drug dosages, irradiation durations and apparatus, and injection methods.

In vivo experiments and treatments, real-time observations, and evaluating the effect of OSMD-based phototherapy are of great importance for studying the mechanism of excessive light exposure and preventing excessive light exposure. However, the use of existing imaging techniques to monitor the production of ROS, especially in the depths of biological tissues, remains a difficult task. PTT is an emerging cancer treatment that relies on the thermal effect of light irradiation to eventually cause cancer cell necrosis. The real-time monitoring of caloric production in PTT has rarely been reported but is essential for clinical applications to guarantee the effect of treatment and prevent tissue overheating damage. Therefore, new materials for imaging and thermal treatment and the real-time detection of thermal changes at tumor sites deep in biological tissues are crucial for the development of PTT.

Currently, several OSMD-based phototrigger therapies have been used in clinic or subjected to trials. However, clinical practice indicates that it is extremely difficult to completely eliminate tumors with monotherapy. Therefore, research has shifted from monotherapy to co-therapy gradually [311]. Multimodal combination therapy may have synergistic effects on distant tumor regression by overcoming hypoxia in deep tumors and local drug limitations and thus present potential applications in clinical transformation. However, its clinical application has bottlenecks. The most important issue is that the biosafety of OSMD-based nanoplatforms involving a variety of materials and treatments has yet to be confirmed. This property will be the key factor affecting the clinical application of OSMD-based nanoplatforms.

## Data Availability

Not applicable.
